# Gene expression profile of the murine ischemic retina and its response to Aflibercept (VEGF-Trap)

**DOI:** 10.1038/s41598-021-94500-1

**Published:** 2021-07-28

**Authors:** Jesús Eduardo Rojo Arias, József Jászai

**Affiliations:** 1 Department of Anatomy, Medical Faculty Carl Gustav Carus, Technische Universität Dresden, Saxony, Germany; 2 Present Address: Wellcome-MRC Cambridge Stem Cell Institute, Jeffrey Cheah Biomedical Centre, Cambridge Biomedical Campus, University of Cambridge, Cambridge, UK

**Keywords:** Transcriptomics, Central nervous system, Visual system, Gene ontology, Microarrays, Angiogenesis, Molecular medicine, Pathogenesis, Diseases, Eye diseases, Retinal diseases, Retinopathy of prematurity

## Abstract

Ischemic retinal dystrophies are leading causes of acquired vision loss. Although the dysregulated expression of the hypoxia-responsive VEGF-A is a major driver of ischemic retinopathies, implication of additional VEGF-family members in their pathogenesis has led to the development of multivalent anti-angiogenic tools. Designed as a decoy receptor for all ligands of VEGFR1 and VEGFR2, Aflibercept is a potent anti-angiogenic agent. Notwithstanding, the molecular mechanisms mediating Aflibercept’s efficacy remain only partially understood. Here, we used the oxygen-induced retinopathy (OIR) mouse as a model system of pathological retinal vascularization to investigate the transcriptional response of the murine retina to hypoxia and of the OIR retina to Aflibercept. While OIR severely impaired transcriptional changes normally ensuing during retinal development, analysis of gene expression patterns hinted at alterations in leukocyte recruitment during the recovery phase of the OIR protocol. Moreover, the levels of Angiopoietin-2, a major player in the progression of diabetic retinopathy, were elevated in OIR tissues and consistently downregulated by Aflibercept. Notably, GO term, KEGG pathway enrichment, and expression dynamics analyses revealed that, beyond regulating angiogenic processes, Aflibercept also modulated inflammation and supported synaptic transmission. Altogether, our findings delineate novel mechanisms potentially underlying Aflibercept’s efficacy against ischemic retinopathies.

## Introduction

Retinopathy of prematurity (ROP) and proliferative diabetic retinopathy (PDR) are prominent microvasculopathies that severely compromise retinal function^1^. Representing leading causes of vision loss worldwide^2^, retinal microvasculopathies are characterised by the emergence of new retinal blood vessels in a process known as neovascularization^3^. Such vessels, however, are immature and leaky. Consequently, nourishment and oxygenation to retinal cells becomes deficient^4^. Together with the accumulation of waste products, the latter ultimately leads to retinal malfunction and vision impairment.

The identification of the vascular endothelial growth factor A (VEGF-A) as a hypoxia-responsive driver of pathological angiogenesis^5^ in the retina^6,7^ and in tumours^8^ led to the development of multiple pharmacological compounds for its neutralization. Despite their efficacy in the treatment of cancer^9,10^ and neovascular retinopathies^11,12^, VEGF-A inhibitors exhibited unforeseen side effects and limitations, including toxicity, short systemic life-times, and limited diffusion across the tissue^3,11,13^. The latter, together with findings suggesting that the placental growth factor (PGF)—another member of the VEGF-family—played a major role in driving pathological neovascularization^14^, boosted interest in the development of multivalent anti-angiogenic therapies^15^. To simultaneously inhibit both PGF and VEGF-A, the decoy receptor Aflibercept (VEGF-Trap, Eylea; hencefort AFL) was created. AFL is a fusion protein formed by the extracellular domains of VEGF receptor 1 (VEGFR1) and VEGFR2 joined to the crystallizable fragment of human IgG^16,17^. Thereby, AFL is capable of neutralizing the ligands of VEGFR1 and VEGFR2^18^ and has received FDA approval for the treatment of age-related macular degeneration (AMD), diabetic macular edema (DME), macular edema following retinal vein occlusion, and background DR^2^. However, AFL has not yet been authorized for use against PDR (off-label only) or ROP, although phase III clinical trials are ongoing for the latter (NCT04004208).

**Figure 1:**
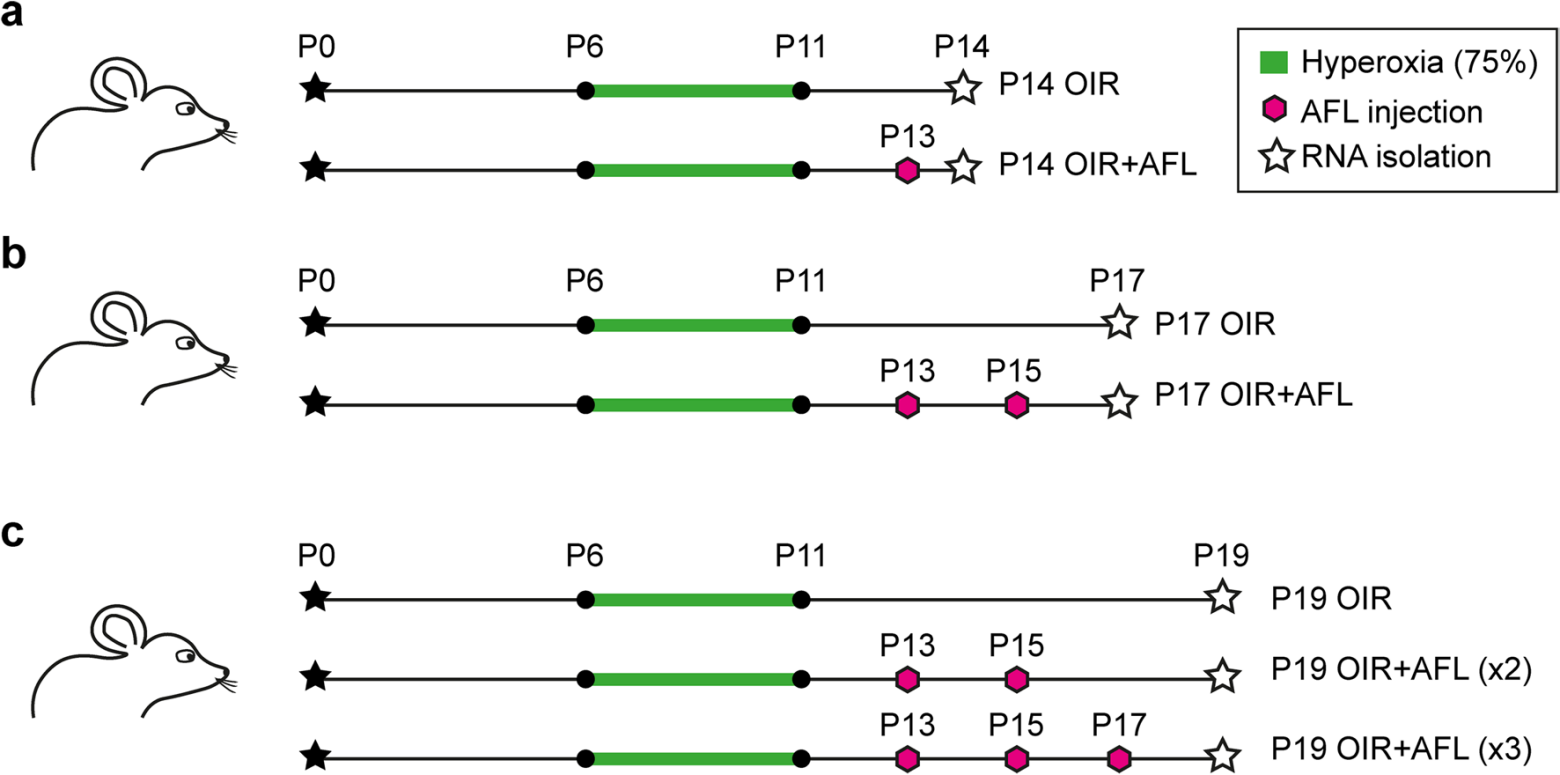
Schematic representation of AFL treatment regimens. (**a**) In the first regimen, mice subjected to the OIR protocol (75% oxygen concentration from P6 to P11) were treated with AFL (25 mg/kg body weight) on P13. Thereafter, on P14, RNA from the retinas of these animals (P14 OIR + AFL) and of non-injected OIR controls (P14 OIR) and normoxic controls (P14 Normoxia; not shown) was collected. (**b**) In a second treatment regimen, mice subjected to the OIR protocol were treated with AFL on P13 and P15 and RNA from their retinas was isolated on P17 (P17 OIR + AFL). Non-injected age-matched OIR littermates (P17 OIR) and normoxic age-matched animals (P17 Normoxia; not shown) were used as controls. (**c**) Finally, mice subjected to the OIR protocol received either 2 or 3 doses of AFL (P19 OIR + AFL (× 2) and P19 OIR + AFL (× 3), respectively) prior to RNA collection on P19. Retinas of non-treated age-matched littermates (P19 OIR) and of normoxic age-matched animals (P19 Normoxia) were used as controls.

Previously, we used the oxygen-induced retinopathy (OIR) mouse model^19^, which represents the most widely studied in vivo model of retinal proliferative microvascular disease^20,21^, to investigate the effects of AFL administration on the ischemic retina^22^. Our study revealed that AFL potently inhibits retinal neovascularization while permitting the concomitant revascularization of the tissue and partially protecting dopaminergic amacrine cells from hypoxic damage. Additionally, transcriptional profiling during the proliferative phase of the OIR protocol (P14 and P17; Fig. 1a, b) revealed that AFL exerts a modulatory effect on the expression of genes associated with angiogenesis and adhesion^22^. Notwithstanding, the molecular mechanisms supporting the endogenous capacity of the murine retina to drive neovascular tuft clearance at later stages remain poorly understood^23^. Moreover, the transcriptional profile of the ischemic retina in response to AFL during the distinct phases of the OIR protocol has not yet been investigated. To address these questions, we extended our transcriptomic analyses to also consider a time-point within the phase of neovascular tuft regression (P19) using two distinct AFL treatment regimens (2 doses vs 3 doses; Fig. 1c). By conducting cross-comparisons between time-points and treatment schedules, our results hint at the involvement of leukocytes during the recovery phase of the OIR protocol (in line with recent reports^24^), and at positive effects of AFL on synaptic transmission in OIR retinas, where its administration seems to protect retinal ganglion cells (RGCs) from hypoxic damage.

## Results

### The OIR protocol profoundly hinders retinal development

The murine retina has been used extensively to study blood vessel formation and endothelial cell (EC) behaviour in health and disease^20^. Here, we used the mouse model of OIR to investigate the transcriptional changes undergone by retinal cells in response to hypoxia and to subsequent anti-angiogenic therapy. To this aim, we administered AFL, a recombinant decoy receptor neutralizing the ligands of VEGFR1 and VEGFR2, at a dose of 25 mg/kg body weight to mice subjected to the OIR protocol. The drug was injected intra-peritoneally (i.p.) following two distinct regimens: either (1) on P13 and P15, or (2) on P13, P15 and P17 (Fig. 1c). At P19, total RNA isolated from whole retinas was analysed by Affymetrix microarrays (see “Methods”). Retinas of age-matched mice reared in normoxia and of OIR littermates not injected with AFL were used as controls.

**Figure 2:**
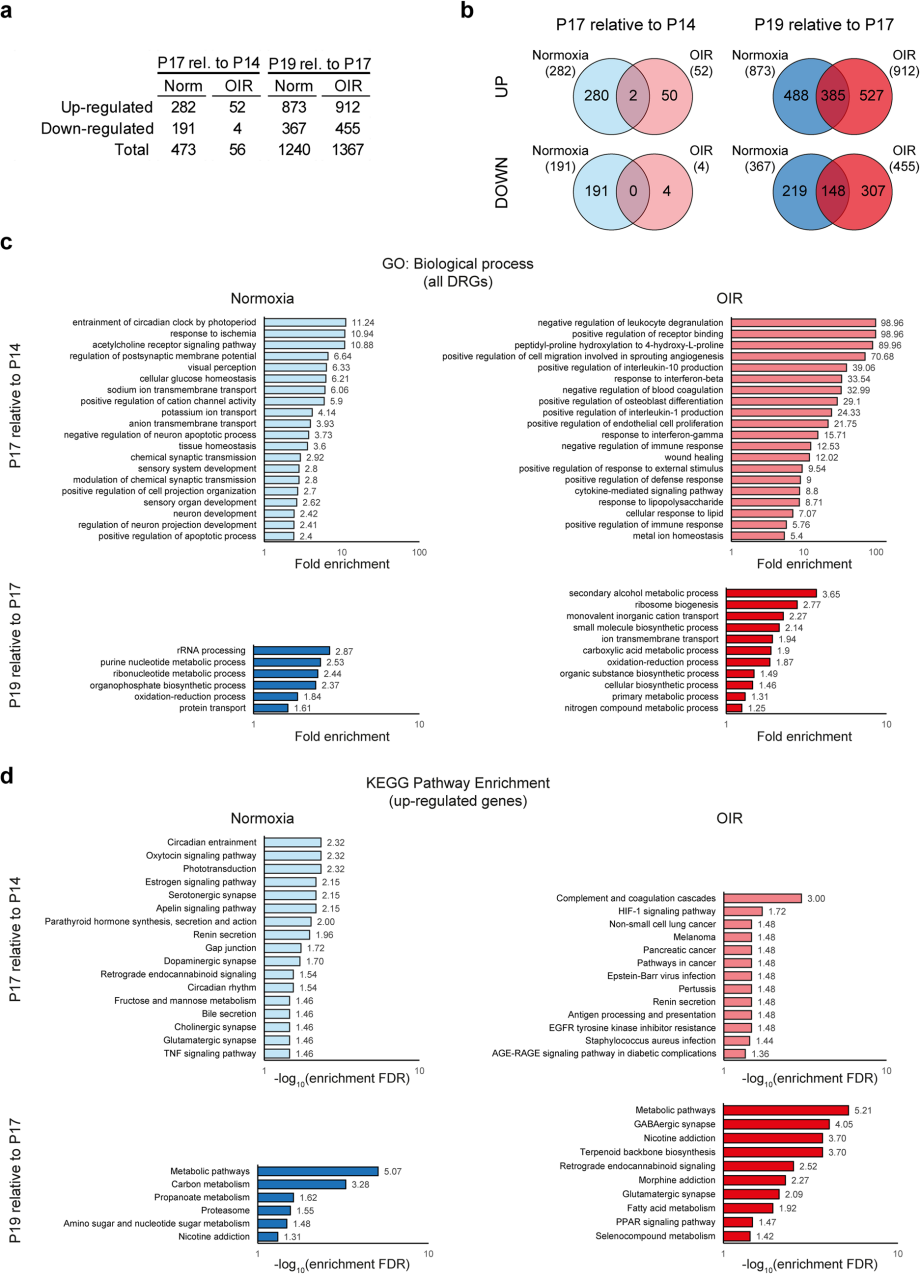
Differential gene expression, GO term enrichment and KEGG Pathway enrichment analyses on retinas of normoxic and OIR mice during early post-natal development. (**a**) Differential gene expression analysis of normoxic and OIR retinas across timepoints. The early transcriptional changes that ensue during normal retinal post-natal development are hindered when mice are subjected to the OIR protocol (left). In contrast, the number of differentially expressed genes between P19 and P17 (right) was higher in OIR retinas than in normoxic controls. (**b**) Venn diagrams of differentially expressed genes in normoxic and OIR retinas on P17 relative to P14 (left) and on P19 relative to P17 (right). While the overlap in both up- (top) and down-regulated genes (bottom) was minimal at the early time frame examined, over 40% of both up- and down-regulated genes in normoxic conditions were also detected in OIR retinas at the later time frame investigated. (**c**) GO term enrichment analysis in the Biological process domain was conducted using all differentially regulated genes detected in Normoxia (left) and OIR (right) retinas at P17 relative to P14 (top) and at P19 relative to P17 (bottom). A fraction of the top enriched GO terms and their fold enrichment are depicted for each condition. (**d**) KEGG pathway enrichment analysis was conducted using all differentially up-regulated genes detected in Normoxia (left) and OIR (right) retinas at P17 relative to P14 (top) and at P19 relative to P17 (bottom). A fraction of the top enriched KEGG Pathways and their − log_10_ enrichment FDR are depicted for each condition.

Previously, we described the transcriptional state of the OIR retina at P14 and P17 after AFL administration^22^ (Fig. 1a,b; GEO accession number: GSE 124956). Here, we build on our previous report to dissect the temporal dynamics of gene expression in normoxic and OIR retinas and to additionally assess the retinal transcriptional state during the recovery phase of the OIR protocol, i.e. at P19, and in response to AFL. First, we addressed whether OIR interfered with the transcriptional changes occurring in the course of normal retinal development. To this aim, we conducted comparisons between the gene expression profiles of P14, P17 and P19 normoxic retinas. The latter revealed moderate changes in transcriptional activity between P14 and P17 (473 differentially regulated genes [DRGs]; fold-change above 1.2 and *P*-value below 0.05), which increased over 2.5 times for the period between P17 and P19 (1240 DRGs; Fig. 2a; Supplementary Tables 1–4). Unexpectedly, only 56 DRGs were identified in OIR retinas in the period between P14 and P17 (Fig. 2a; Supplementary Tables 5–6). In contrast, 1367 DRGs were found between P17 and P19 in OIR retinas (Supplementary Tables 7–8), which represents ~ 10% more than in normoxic samples during the same time frame (Fig. 2a). Surprisingly, only 2 of the 282 differentially up-regulated genes from P14 to P17 in normoxia (< 1%) were also up-regulated in OIR mice (~ 4%), with none of the down-regulated genes being found in both OIR and normoxic retinas during this period (Fig. 2b, left). Conversely, ~ 44% (385/873) of the up-regulated genes and ~ 40% (148/367) of the down-regulated genes found in normoxic tissues between P17 and P19 were also detected as differentially expressed in OIR retinas during this time window (Fig. 2b, right). The latter accounted for ~ 42% and ~ 32.5% of all differentially up- and down-regulated genes in OIR retinas, respectively (912 up, 455 down). These observations suggested a delay in the maturation process of the retinal tissue as a consequence of the OIR protocol. In this context, gene ontology (GO) term enrichment analysis (Fig. 2c, top)^25–27^ using genes differentially expressed in normoxic retinas at P17 relative to P14 identified terms associated to retinal development and visual perception (e.g. *visual perception*, *sensory organ development*), potentially supported by enhanced ion transport (e.g. *potassium ion transport*), and neuronal and synaptic maturation (e.g. *chemical synaptic transmission*, *neuron development*). In contrast, GO terms enriched in OIR retinas at P17 relative to P14 were primarily associated to injury repair (e.g. *wound healing*), inflammation (e.g. *response to interferon-gamma*), hypoxia (*peptidyl-proline hydroxylation to 4-hydroxy-L-proline*), and angiogenesis (e.g. *positive regulation of cell migration involved in sprouting angiogenesis*). In line with these findings, a recent study reported that sterile inflammation and the recruitment of neutrophils are both essential for the clearance of neovascular tufts from the OIR retina^24^. Meanwhile, GO terms enriched at P19 compared to P17 were mainly related to metabolic changes in both normoxic and OIR retinas (Fig. 2c, bottom). An unexpected finding at first sight was the enrichment of the GO term “response to ischemia” in the set of DRGs identified in normoxic retinas from P14 to P17 (Fig. 2a). Nonetheless, moderate hypoxia is known to be required for retinal development and vascularization^28^. Among the genes associated to this GO term, *Egr1* encodes the Early growth response 1 transcription factor, which regulates the expression of multiple genes, including the Hypoxia inducible factor 1 subunit alpha (HIF-1α)^29^.

To complement our GO term enrichment analysis, we conducted KEGG pathway enrichment analyses. Focusing solely on up-regulated genes, we identified KEGG pathways associated to synaptic maturation (e.g. *glutamatergic synapse*) as enriched from P14 to P17 in normoxic retinas (Fig. 2d, top). In comparison, the KEGG pathways enriched during this timeframe in OIR retinas were associated to hypoxia (*HIF-1 signaling pathway*), cancer (*e.g. melanoma*), response to infection (*e.g. staphylococcus aureus infection*) and diabetes (*AGE-RAGE signalling pathway in diabetic complications*). In agreement with our GO term enrichment analysis, KEGG pathway enrichment analysis also revealed that most changes between P17 and P19 were metabolic, both in normoxic and OIR retinas (Fig. 2d, bottom). Together, the findings of these analyses suggest that the maturation of retinal neurons, occurring during a limited post-natal time window, might be severely impaired in the OIR retina. In this context, we have previously shown that the number of dopaminergic amacrine cells is indeed significantly reduced as a consequence of the OIR protocol, with numbers failing to reach normoxic levels even in young adult animals^22^.

### Genes associated to angiogenesis, metabolism, and leukocyte migration are regulated in the post-P17 recovery phase of the OIR protocol

**Figure 3:**
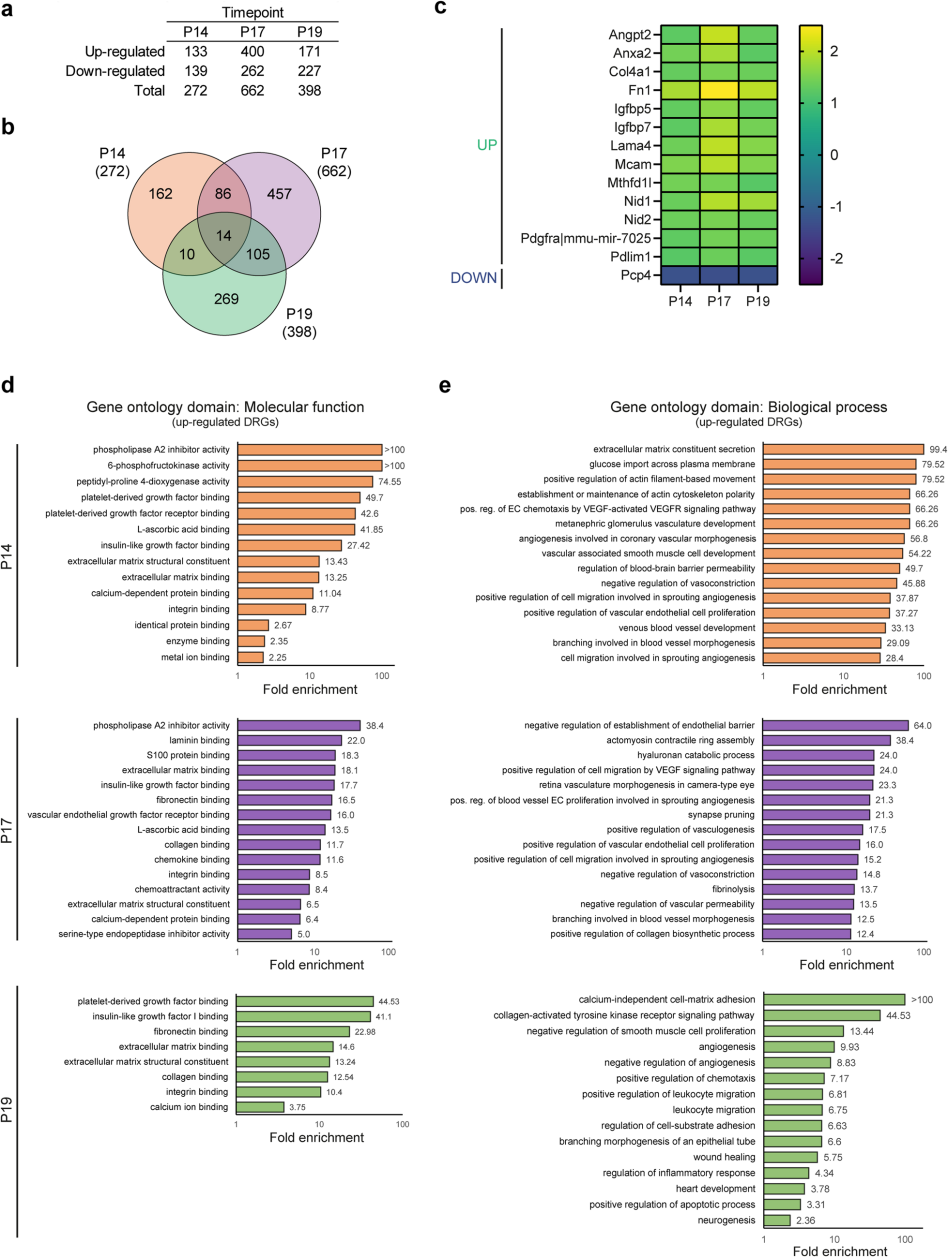
Differential gene expression and GO term enrichment analyses on retinas of OIR mice relative to retinas of normoxic age-matched controls at each of the assessed timepoints. (**a**) Differential gene expression analysis on OIR retinas compared to normoxic age-matched controls across timepoints. Important deviations in gene expression profiles are detected, the most prominent of which occurs at P17. (**b**) Venn diagram of differentially expressed genes in OIR retinas relative to normoxic age-matched control samples. Only 14 transcripts were identified as differentially regulated at all three timepoints. (**c**) Heatmap depicting the fold-change expression of the 14 differentially regulated genes identified in (**b**), of which 13 were up-regulated and 1 was down-regulated. Noteworthy, the expression levels of up-regulated genes remained high throughout time, while the expression of the down-regulated transcript remained low at all timepoints examined. (**d**) GO term enrichment analysis in the Molecular function domain was conducted using all differentially up-regulated genes identified in OIR retinas relative to normoxic retinas at each time point (P14, top; P17, middle; P19, bottom). A subset of all enriched GO terms and their fold enrichment are depicted. (**e**) GO term enrichment analysis in the Biological process domain was conducted using all differentially up-regulated genes identified in OIR retinas relative to normoxic retinas at each time point (P14, top; P17, middle; P19, bottom). A subset of all enriched GO terms and their fold enrichment are depicted.

Next, we analysed how the transcriptional profiles of OIR and normoxic retinas diverged. We identified 272, 662, and 398 DRGs in OIR retinas compared to normoxic controls at P14, P17, and P19, respectively (Fig. 3a; Supplementary Tables 9–14). The latter suggests that the transcriptional response of the murine retina to the OIR protocol is most pronounced at P17. Interestingly, however, only 14 DRGs were detected in response to OIR at all three time-points (Fig. 3b). Among them, 13 genes were up-regulated and 1 was down-regulated (Fig. 3c). Most of the up-regulated genes encoded either components of the vascular wall (*Col4a1, Fn1*, *Lama4*, *Mcam*, *Nid1*, *Nid2*) or proteins relevant for cell-extracellular matrix interactions (*Anxa2*, *Pdlim1*). Whereas the synthesis and deposition of basement membrane components such as collagen IV (of which a component is encoded by *Col4a1*) by ECs represent crucial processes in blood vessel formation and maturation^30,31^, *Anxa2* expression is known to be regulated by VEGF and has been previously reported to be elevated in OIR retinas, with its knockdown supressing retinal neovascularization^32^. Further, the up-regulated expression of *Angpt2* at all examined time-points might be of key relevance as Angiopoietin-2 (Ang2), the protein it encodes, has been detected in high levels in the vitreous of individuals with DR^33^ and agents for its blockade (e.g. Faricimab) are currently undergoing clinical trials. Additionally, the PDZ and LIM domain protein 1 (encoded by *Pdlim1*) has been recently reported to play a role in the assembly of stress fibres and to be potentially involved in cell migration^34^, a crucial process for the formation of neovascular tufts. While the role of mir-7025 in the context of retinopathy requires investigation, the only gene detected as down-regulated in OIR retinas relative to normoxic controls at all three examined time-points was *Pcp4*. Encoding Purkinje Cell Protein 4, *Pcp4* expression has been reported in ganglion, amacrine and horizontal cells^35^. Thus, our findings hint at a potentially detrimental effect of OIR on these retinal cell populations. Further, the expression of the Insulin-like growth factor-binding proteins 5 and 7 (*Igfbp5* and *Igfbp7)* was higher in OIR than in normoxic retinas (Fig. 3c). ECs are known to store the protein encoded by *Igfbp7*, Angiomodulin, together with Ang2 and others in Weibel-Palade bodies, storage organelles unique to vascular ECs that deliver mediators of inflammation and hemostasis in response to certain stimuli^36^. Moreover, Angiomodulin is also a marker of cancer vasculature known to be regulated by VEGF^37^ and to modulate neoangiogenesis in response^38^. In line with these reports, elevated mRNA expression of *Igfbps*, including *Igfbp5* and *Igfbp7*, has been previously found in OIR retinas, specifically within neovascular tufts^39^. Notwithstanding, the specific function of these molecules at this location is unclear and requires further investigation.

To determine whether gene sets associated to specific cellular functions were differentially expressed in OIR retinas relative to normoxic controls, we conducted GO term enrichment analysis at each time-point. Whereas the most strongly enriched terms in the molecular function domain at P14 (Fig. 3d, top) were associated to metabolism (e.g. *6-phosphofructokinase activity*), most were related to the binding of cellular and extracellular components (e.g. *extracellular matrix binding*, *integrin binding*). At this time-point, GO terms enriched in the biological process domain (Fig. 3e, top) were primarily related to angiogenesis (e.g. *venous blood vessel development*). The latter, however, are likely linked to pathological aspects of blood vessel formation as enriched GO terms also included the negative regulation of vasoconstriction and the regulation of blood–brain barrier permeability. In agreement, we previously reported hyperpermeability and a higher number of dilated vessels in the OIR retina than in normoxic age-matched controls^22^. Notably, the GO term “positive regulation of endothelial cell chemotaxis by VEGF-activated VEGFR signaling pathway” was also enriched, evidencing the role of VEGF in the pathogenesis of OIR.

At P17, GO terms enriched in the molecular function domain (Fig. 3d, middle) were similar to those detected at P14, albeit including terms associated to immune cell recruitment (e.g. *chemoattractant activity*) and VEGF signalling (*vascular endothelial growth factor receptor binding*). GO terms enriched in the biological function domain at this time-point also partially overlapped with those detected at P14 (Fig. 3e, middle), and were primarily related to blood vessel formation, vasoconstriction, and vascular permeability. GO terms enriched at P17 but not at P14 included “positive regulation of collagen biosynthetic process”, “fibrinolysis” and “synapse pruning”. The latter points in the direction of a compromised neuronal network and is in line with our findings in the developmental context (Fig. 2).

Although most of the GO terms identified as enriched at P19 within the molecular function domain had already been detected at P17, previously unidentified terms included “insulin-like growth factor I binding” and “platelet-derived growth factor binding” (Fig. 3d, bottom). Meanwhile, many of the GO terms enriched at P19 within the biological process domain (Fig. 3e, bottom) were, as observed at P17, related to angiogenesis. Additionally, resembling the results of our analysis across time-points (Fig. 2), terms associated to inflammation, injury response and neuronal development were detected as enriched (e.g. *regulation of inflammatory response*, *wound healing, neurogenesis*). Remarkably, enriched terms also included some related to leukocyte recruitment and to increased apoptosis. These findings are in agreement with previous reports describing a decrease in the number of Foxp3^+^ Regulatory T cells occurring concomitantly with the emergence of microvascular aberrations in the OIR retina^40^, as well as a central role for neutrophils in repairing such aberrations by promoting the apoptosis-mediated clearance of senescent ECs within neovascular tufts^24^. In particular, 6 genes (*Pecam1*, *Ednra*, *Mstn*, *Cx3cr1*, *Mmp14*, *Nckap1l*) belonging to the “positive regulation of leukocyte migration” GO term were detected among those differentially up-regulated in P19 OIR retinas relative to age-matched controls. Meanwhile, 8 genes belonging to the “leukocyte migration” term were detected, including 5 not associated to the term “positive regulation of leukocyte migration”, namely *Ccl3*, *Itgb1*, *Fcer1g*, *Cd34* and *Itga6*. Additionally, 9 genes were associated to the term “positive regulation of chemotaxis”, including 7 not associated to neither of the two previous GO terms*: Pdgfrb*, *Sema7a*, *Padi2*, *Angpt2*, *Tubb2b* and *Fn1*.

Among these genes, *Pecam1* is known to be required for leukocyte transendothelial migration under inflammatory conditions^41^, *Mstn* was recently shown to be expressed in bloodstream-derived leukocytes^42^, and integrin β_1_ (encoded by *Itgb1*) was recently described to mediate the adhesion of monocytes to liver sinusoidal ECs and thereby contribute to hepatic inflammation^43^. Further, detecting not only *Itgb1* but also *Sema7a* as up-regulated is remarkable given that endothelial Semaphorin 7A is known to promote neutrophil migration during hypoxia^44^ and that vascular Semaphorin 7A up-regulation has been shown to promote atherosclerosis through endothelial β1 integrin^45^. Similarly, monocyte/macrophage-derived matrix metallopeptidase 14 (encoded by the *Mmp14* gene) has been reported to modulate monocyte infiltration in a context-dependent manner^46^ while CC chemokine ligand 3 (encoded by *Ccl3*) is known to induce neutrophil chemotaxis under conditions of acute inflammation^47^. Meanwhile, knockdown of *nckap1l* in zebrafish has recently been reported to reduce neutrophil migration^48^, and its upregulation might thus promote neutrophil recruitment. Hence, an increased presence of leukocytes in the OIR retina at P19 is likely. Further, up-regulation of *Cx3cr1* might be linked to the significant increase in the number of microglial cells previously reported to occur in the OIR retina^49^, although it is surprising that the expression of this gene was not up-regulated neither at P17 nor at P14. Considering that Cx3cr1 depletion has been associated to an accelerated progression of retinopathy in the rat model of streptozotocin-induced diabetes^50^, its up-regulation during the post-P17 phase of the OIR protocol might also play a relevant role in recovery. Together, these findings suggest that multiple genes encoding proteins with pronounced, and in certain cases complementary, regulatory effects on leukocyte migration are upregulated in the OIR retina at P19.

### Genes regulated by AFL are involved in angiogenesis, immune cell recruitment, and neurotransmission

**Figure 4:**
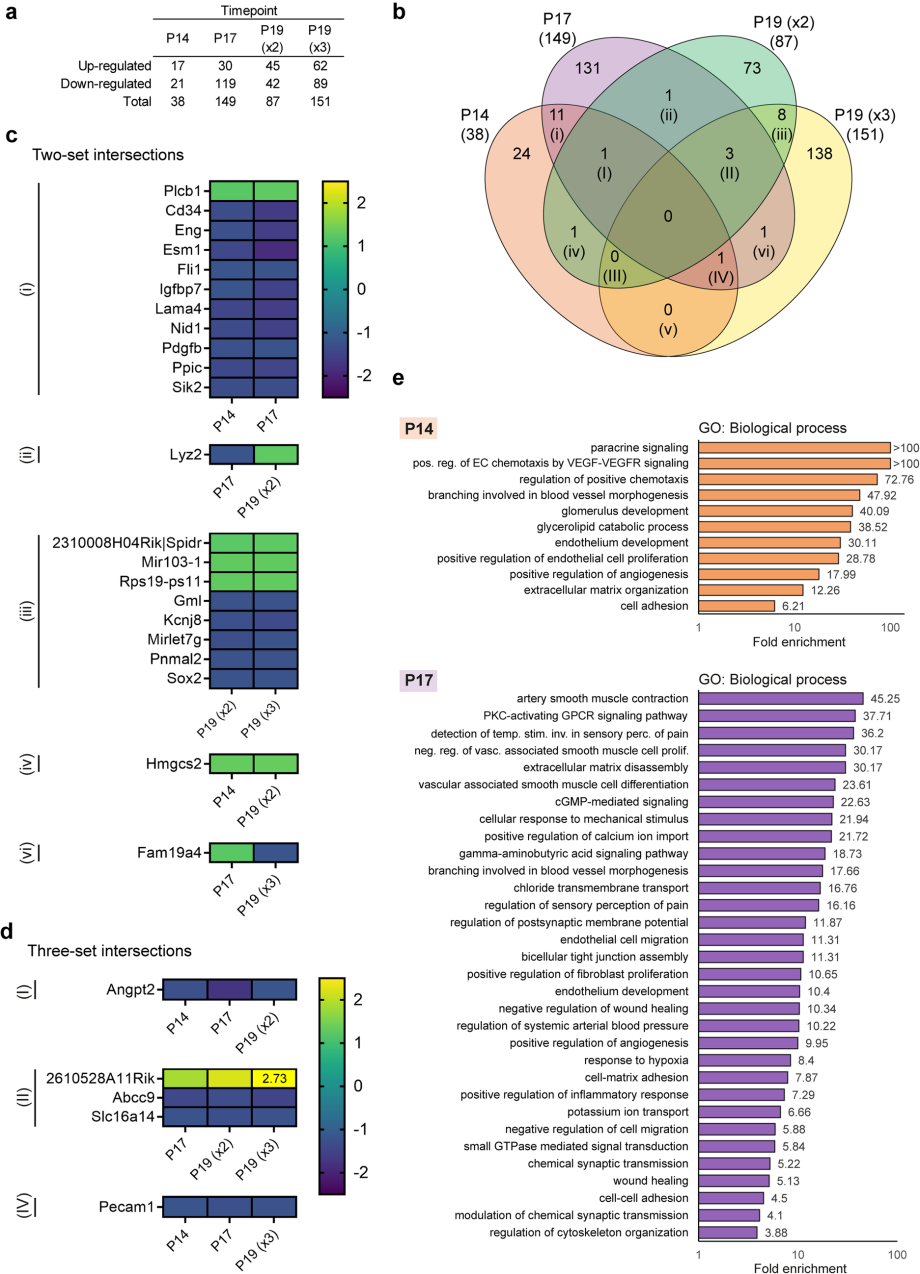
Differential gene expression and GO term enrichment analyses on retinas of OIR + AFL mice relative to retinas of non-injected OIR age-matched controls. (**a**) Differential gene expression analysis on retinas of OIR mice that received AFL compared to retinas of OIR age-matched mice that did not at each investigated timepoint. A similar impact on the differential regulation of gene expression by AFL is detected after 2 doses of AFL at P17 (149 DRGs) and after 3 doses at P19 (151 DRGs). (**b**) Venn diagram depicting the overlap in differentially regulated genes identified at each timepoint in retinas of OIR mice treated with AFL relative to retinas of age-matched OIR controls. (× 2) refers to 2 AFL doses and × 3 to 3 AFL doses. (**c**) Heatmaps depicting the fold-change in expression of differentially regulated genes identified in two conditions. The specific overlap regions are referenced to the Venn diagram in (**b**) by the roman numerals i-vi. Note that no genes were identified to be differentially regulated in (v), i.e. both at P14 and P19 (× 3). (**d**) Heatmaps depicting the fold-change in expression of differentially regulated genes identified in three conditions. The specific overlap regions are referenced to the Venn diagram in (**b**) by the roman numerals I-IV. Note that no genes were identified to be differentially regulated in (III), i.e. both at P14, P19 (× 2) and P19 (× 3). In (II) the fold-change of *2610528A11Rik* is written on the heatmap for the P19 (× 3) time-point. (**e**) GO term enrichment analysis in the Biological process domain was conducted using all differentially regulated genes identified in AFL-treated OIR retinas relative to non-treated OIR control retinas at P14 (top) and P17 (bottom). A subset of all enriched GO terms and their fold enrichment are depicted.

To shed light on the transcriptional processes mediating the capacity of anti-angiogenic therapy to counteract pathological neovascularization and reduce hypoxia-induced damage in the retina, OIR animals were treated with AFL (treatment regimens shown in Fig. 1). AFL administration led to the differential regulation of 38 and 149 genes at P14 and P17, respectively (Fig. 4a; Supplementary Tables 15–18). Meanwhile, 87 and 151 DRGs were detected at P19 in retinas of OIR mice that received AFL on two and three occasions (P19 AFLx2 and P19 AFLx3), respectively (Fig. 4a; Supplementary Tables 19–22). Although no single DRG was detected in all treatment regimens, 27 genes were differentially regulated at least in two of them, and 5 of these in three regimens (Fig. 4b).

Among the DRGs detected in two regimens, those found at P14 and P17 largely overlapped with those that we previously reported^22^ (Fig. 4c, [i]), confirming that the normalization strategy used for this study did not lead to dramatic deviations from our prior findings. Interestingly, while retinas of animals that received two doses of AFL expressed lower levels of *Lyz2* than those of OIR non-injected littermates at P17, this gene was up-regulated at P19 in retinas of mice that followed the same treatment regimen (Fig. 4c, [ii]). *Lyz2* has recently been proposed as a marker for activated microglia in the context of retinal degeneration^51^, with a previous study revealing that the activation status of microglial cells is more important than their density in the emergence of OIR-driven microvascular aberrations^52^. Considering that proangiogenic *Lyz2*-expressing mononuclear cells are selectively recruited to sites of retinal neovascularization in response to VEGF-A and Semaphorin 3A^53^, we hypothesized that the initial reduction and subsequent up-regulation of *Lyz2* could potentially reflect the kinetics of VEGF-A neutralization in animals that received two doses of AFL. However, upon closer inspection, we noticed that AFL administration led to an increase in the expression levels of *Lyz2* from P14 to P17 which was of the same magnitude as that observed in retinas of OIR animals that did not receive AFL during the same period (Supplementary Fig. S1a). In contrast, the expression dynamics of this factor in OIR retinas showed a pronounced peak at P17 that by P19 returned to the levels observed at P14. As the levels of this transcript increased progressively over-time in retinas of AFL-treated mice, its identification as differentially regulated seems to be mainly a consequence of the marked changes in its expression dynamics in the OIR retina, which might in turn reflect a sharp increase in the number of mononuclear cells present in the tissue at P17 as a key step towards neovascular tuft clearance^24^. In line with this hypothesis, *Fam19a4* encodes the Family with sequence similarity 19 (chemokine (C–C motif)-like) protein, a secreted molecule that modulates macrophage chemotaxis and activation^54^ whose expression levels were higher in OIR + AFL than in OIR retinas at P17 but lower in OIR + AFLx3 retinas than in OIR(Fig. 4c, [vi]). As in the case of *Lyz2*, examination of the expression dynamics of *Fam19a4* revealed a marked capacity of the OIR retina to endogenously regulate the levels of this gene (Supplementary Fig. 1b). Interestingly, *Lyz2* expression in normoxic and OIR + AFL retinas followed a similar trend, although its levels remained slightly lower in AFL-treated retinas than in controls. Thus, these two genes might serve as important mediators of the retinal endogenous capacity to recover from OIR; the molecular machinery regulating their expression and function in this context remains to be further investigated.

Unexpectedly, only 11 genes were differentially regulated at P19 in response to both two and three AFL doses (Fig. 4b, [iii] and [II]). DRGs detected in only these two conditions (Fig. 4c [iii]) encode an inwardly rectifying potassium channel (*Kcnj8*; Kir6.1 channel), a transcription factor (*Sox2*), a microRNA (*Mirlet7g*), the Glycosylphosphatidylinositol-anchored molecule-like (*Gml*), and an uncharacterized protein (*Pnmal2*). *Kcnj8* mutations have been linked to excessive retinal vessel formation and increased vascular tortuosity in retinas of children and adults affected with Cantú syndrome, respectively^55^, and Kir6.1 expression has been detected in human retinal pigment epithelium (RPE)^56^. Further, expression of the microRNA (miR) let-7 g is downregulated in the heart of mice after coronary occlusion in vivo and in neonatal rat ventricular myocytes subjected to hypoxia in vitro, with its ectopic expression preventing hypoxia-dependent caspase activation and death in both settings^57^. Similarly, hypoxia has been shown to increase the expression of Sox2 in mesenchymal stem cells^58^, with higher oxygen levels leading to its reduced expression in embryos *in vitro*^59^. Therefore, the reduced expression levels of *Kcnj8, Mirlet7g*, and *Sox2* in response to AFL might evidence its beneficial effects on retinal oxygenation, with their functional relevance in the OIR retina requiring further investigation. Meanwhile, *Gml* is speculated to be a p53 target involved in the apoptotic pathway and in cell-cycle regulation^60^. Yet, its relevance to retinal disease, as that of the uncharacterized *Pnmal2*, remains unknown.

Three genes were up-regulated in both P19 OIR + AFLx2 and P19 OIR + AFLx3 retinas compared to OIR controls (Fig. 4c [iii]): *2310008H04Rik* (also *Spidr*), *Mir103-1*, and *Rps19-ps11*. The functions of *2310008H04Rik*, encoding a scaffolding protein involved in DNA repair, in the context of retinal disease are presently unknown. Meanwhile, *Rps19-ps11* encodes a pseudogene of the ribosomal protein S19 (Rps19). Although pseudogenes are presumed to be non-functional as a consequence of mutations and truncation due to premature stop codons, some are protein-coding^61^. In this sense, knockout of *Rps19* is pre-implantation lethal in mice^62^ and its knockdown has been shown to result in stagnant blood cells as a consequence of circulatory defects in zebrafish (*Danio rerio*)^63^. Notably, by binding the macrophage migration inhibitory factor (MIF), Rps19 reduces the adhesion of monocytes to ECs^64^, which is a key step in the inflammatory response. In turn, *miR103-1*, whose expression is downregulated in RPE cells in response to hypoxia^65^, binds the nuclear factor-κB (NFκB) Interacting LncRNA (NKILA), whose high levels drive the up-regulation of NFκB, lead to neuronal cell death and protect RPE cells from hypoxic damage^66^. Hence, the upregulation of these genes at P19 in response to AFL might be relevant for the regeneration of the retinal microvascular network, to modulate inflammation, and to protect retinal cells from hypoxia.

Interestingly, only one DRG was up-regulated in response to AFL both at P14 and P19 (× 2) (Fig. 4c [iv]), namely *Hmgcs2,* which encodes the ketogenesis rate-limiting enzyme 3-hydroxy-3-methylglutaryl-Coenzyme A synthase 2 (mHS). *HMGCS2* is highly expressed by primary human foetal RPE cells in vitro^67^ and mHS is one of three mitochondrial enzymes often inactivated in cancer^68^. Moreover, the product of mHS, β-hydroxybutyrate, has a role not only in cellular energetics but also as an agonist of the cell surface receptor GPR109A, a tumour suppressor relevant to apoptosis^68^. As the metabolic transcriptome of retinal ECs undergoes profound changes in pathological conditions^69^, the relevance of *Hmgcs2* as a possible regulator of EC apoptosis in the OIR retina should be addressed in future studies.

Five genes were identified as differentially regulated in response to AFL in three treatment regimens (Fig. 4d). At P14, P17 and P19(× 2), AFL significantly down-regulated the expression of *Angpt2* (Fig. 4d, [I]), whose levels were also higher in OIR than in normoxia (Fig. 3c). The latter suggests that, by reducing the expression of *Angpt2* and neutralizing the ligands of VEGFR1 and VEGFR2 simultaneously, AFL might be a more effective anti-angiogenic agent than previously recognized. At P17, P19(× 2) and P19(× 3), three genes were differentially regulated in response to AFL (Fig. 4d[II]). Encoded by *Slc16a14*, the monocarboxylate transporter 14 (MCT14) has been previously detected in mouse kidneys and brain^70^, with its expression recently reported to be confined to the endothelial compartment in the central nervous system^71^. Notwithstanding, MCT14 remains an orphan transporter with unknown functions or substrate specificities^72^. Similarly, the role of *2610528A11Rik* in the retina is currently unknown. However, its human ortholog, chromosome 10 open reading frame 99 (C10ORF99), is structurally similar to chemokines of the CC family and modulates the infiltration of T cells to skin transplants in mice^73^. On this basis, we hypothesize that the upregulation of *2610528A11Rik* in response to AFL might be relevant to regulate leukocyte recruitment to the OIR retina. Meanwhile, *Abcc9*, encoding the ATP-binding cassette subfamily C member 9 protein, has been reported as down-regulated in murine OIR retinas^74^ and, similar to *KCNJ8*, gain-of-function mutations in *ABCC9* have also been reported in Cantú syndrome^75,76^. As both *Kcnj8* and *Abcc9* encode subunits of ATP-sensitive potassium channels, which have been linked to enhanced retinal resistance against severe ischemic insults^77^, their AFL-induced down-regulation at P17 and P19 might be a consequence of improved retinal oxygenation.

At P14, P17 and P19(× 3), AFL administration led to the reduced expression of *Pecam1* (Fig. 4d, [IV]), which encodes the platelet endothelial cell adhesion molecule-1 (or CD31). In PECAM-1-deficient mice, retinal vascular density is reduced as a consequence of increased EC apoptosis^78^. Interestingly, when subjected to the OIR protocol, these animals also display reduced retinal neovascularization in spite of VEGF levels being similar to those of wild-type control retinas^78^. Thus, its down-regulation by AFL might promote apoptosis in neovascular tufts.

GO term enrichment analysis using genes differentially regulated in response to AFL revealed the robust modulatory effects that this agent exerts on angiogenesis even 24 h after the first dose (Fig. 4e, top). Meanwhile, GO terms enriched in the biological process domain at P17 (Fig. 4e, bottom) were associated to angiogenesis, smooth muscle cell function (e.g. contraction, proliferation), the extracellular matrix (e.g. disassembly, cell–matrix adhesion), response to mechanical stimuli, ion transport (e.g. potassium, chloride), neurotransmission, and synaptic maturation. Among the latter, the enriched GO term “chemical synaptic transmission” included the genes *Gabra4*, *Tax2*, *P2rx2*, *Gabrb1*, *Glra1*, *Shc3*, *Cplx1*, *Plat*, *Drd4*, and *Gabrg3*. From these genes, only 2 (*Drd4* and *Plat*) had higher expression in OIR than in normoxic retinas, with 7 being lower and one (*Tac2*) non-significant (Supplementary Fig. S2). *Plat* encodes a serine protease secreted by endothelial, neuronal, microglial and astrocytic cells which is crucial for fibrinolysis^79^ and which, in cancer, plays a role in vascular remodelling^80^. Thus, its role might be more linked to angiogenesis than to synaptic transmission per se. Meanwhile, *Drd4* encodes the Dopamine receptor D4, whose expression is known to be induced by hypoxia^81^ and to reach a peak around P12 in the rat retina^82^, which correlates with photoreceptor maturation in this species.

Further, it was striking to detect three distinct subunits of the Gamma-aminobutyric acid (GABA) receptor Type A (GABA_A_) as differentially down-regulated in OIR retinas, with two of them fully and one of them partially restoring their expression levels relative to normoxia in response to AFL. GABA_A_ receptors are expressed in RGCs, where their blockade has been shown to reduce oxidative stress-induced death^83^. Whereas this finding by itself could suggest that RGCs engage in a protective behaviour to reduce hypoxia-associated damage and death, the reduced expression levels of *Glra1*, which encodes the subunit alpha-1 of the Glycine receptor (also expressed in RGCs^84,85^), may instead indicate that RGC functionality and survival is compromised as a consequence of OIR and partially rescued by AFL administration. Finally, among the 10 genes associated to the chemical synaptic transmission term, *P2rx2*, which encodes a subunit of a ionotropic receptor expressed in starburst amacrine cells^86^, was the most significantly down-regulated gene in OIR relative to normoxic retinas and subsequently up-regulated in response to AFL at P17. No GO terms in the biological process domain were detected as enriched in P19(AFLx2) or P19(AFLx3) retinas. Overall, our findings suggest that, beyond modulating angiogenic processes in the OIR retina while exerting regulatory effects on apoptosis and ion transport, AFL also aids in preventing hypoxic damage to retinal neurons, with changes in gene expression suggesting the protection of RGCs and amacrine cells.

### The genes endogenously modulated by the OIR retina between P17 and P19 overlap only marginally with those regulated by AFL at P14 and P17

**Figure 5:**
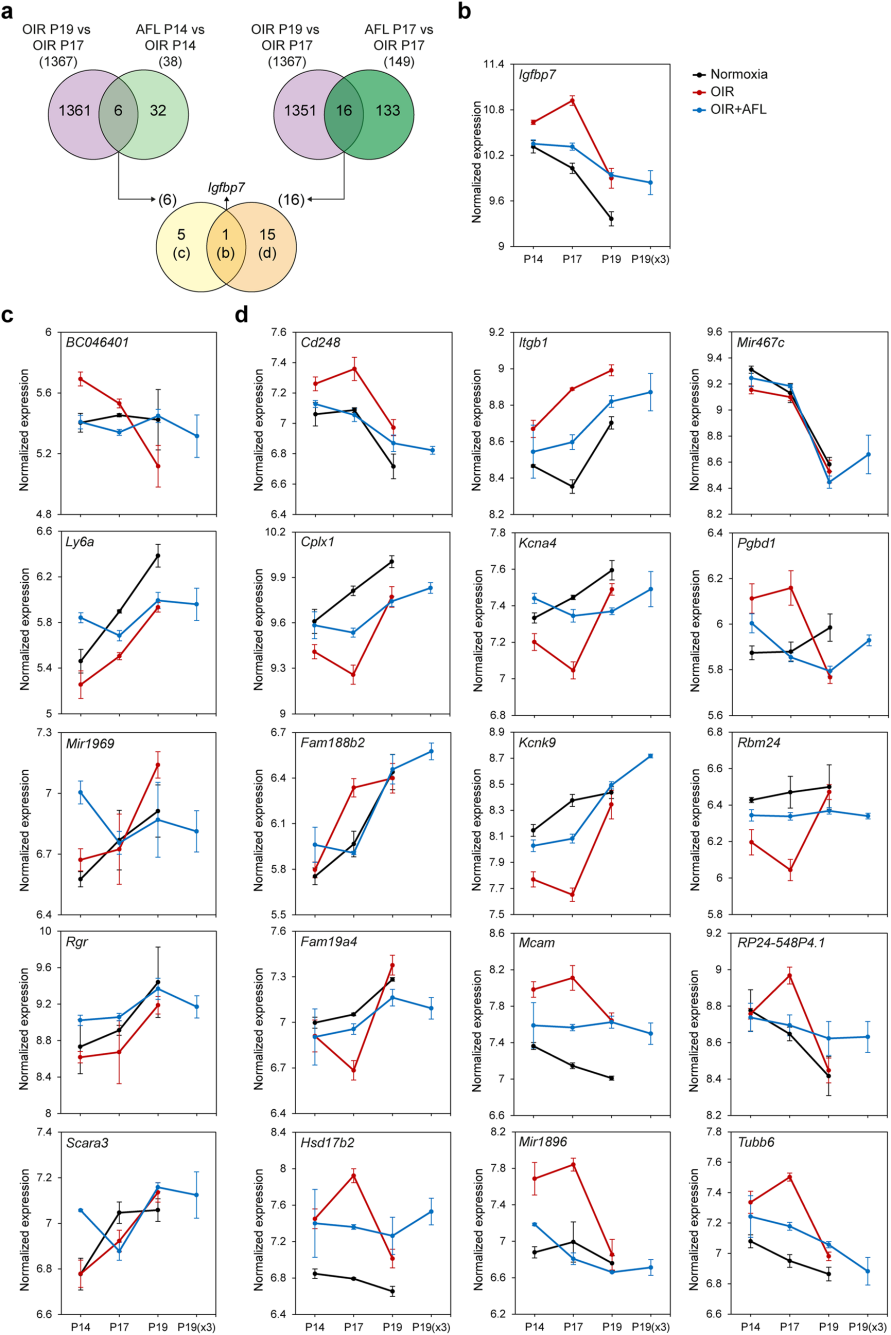
Genes differentially expressed both during the post-P17 recovery phase of the OIR protocol and in response to AFL administration either at P14 or at P17. (**a**) Venn diagrams depicting the overlap in differentially regulated genes identified from P17 to P19 in OIR retinas and either in P14 (left) or P17 (right) OIR retinas after AFL administration. The two sets of genes overlapping in these comparisons were compared to each other, with only one common gene identified (middle). (**b–d**) Normalized expression levels of the genes identified in the overlaps of the Venn diagrams in (**a**) at each time-point (P14, P17, P19) and condition (Normoxia, OIR, OIR + AFL). The additional P19(× 3) time-point refers to retinas of OIR animals that received 3 doses of AFL and were examined at P19. Error bars in (**b–d**) depict ± 1 S.E.M.

To investigate whether the transcriptional changes undergone by the retina to repair the microvascular aberrations arising as a consequence of the OIR protocol overlap with those modulated by AFL, we compared the genes differentially regulated between P17 and P19 in OIR retinas with those modulated by AFL administration either at P14 or at P17. Of the 1367 DRGs identified in OIR from P17 to P19, AFL had an effect on the expression of only 6 of them at P14 and on 16 of them at P17, with *Igfbp7* being the only gene common to both stages (Fig. 5a). While the elevated expression of *Igfbps* had been reported in other studies on the OIR model^39^, it was so far unknown that AFL can maintain the expression levels of *Igfbp7* in the OIR retina much closer to those observed in normoxic age-matched tissues and that this gene is significantly down-regulated during the recovery phase of the OIR protocol (Fig. 5b).

Of the 5 remaining transcripts downregulated both by AFL at P14 and between P17 and P19 in OIR, AFL only restored the expression level of one of them, namely BC046401; for the other 4 transcripts, AFL administration led to an expression level significantly higher than in OIR or normoxia (Fig. 5c). Among these genes, the protein encoded by *Scara3*, Scavenger Receptor Class A Member 3 (also known as Cellular Stress Response Gene Protein, CSR), has been shown to protect cells from oxidative stress by scavenging oxidative molecules and harmful products of oxidation^87^, while Lymphocyte antigen 6A-2/6E-1 (also known as Stem cells antigen-1, Sca-1), encoded by *Ly6a*, marks a population of multipotent cells in the neonatal murine retina^88^ and of bone marrow stem cells which differentiate into glial and microglial cells that help preserve visual function in a mouse model of retinal ischemia/reperfusion^89^. Importantly, as Ly6G inhibition was recently reported to impair the endogenous capacity of the murine OIR retina to clear neovascular tufts after P17 by depleting it from neutrophils^24^, the up-regulation of *Ly6a* we observed in P14 OIR + AFL retinas relative to P14 OIR ones might reflect an increased presence of neutrophils with the capacity to promptly destroy such tufts. Thus, these findings suggest that AFL may protect the retina from hypoxia-induced damage by increasing the cell’s capacity to tolerate oxidative stress while supporting the presence of microglial cells and tuft-clearing neutrophils.

From the 15 genes differentially regulated both in P19 OIR retinas relative to P17 OIR ones and in P17 OIR + AFL retinas relative to OIR ones at P17 but not at P14, the expression levels of 14 of them were either fully (*Cd248*, *Fam188b2*, *Fam19a4*, *Kcna4*, *Mir1896*, *Pgbd1*, *RP24-548P4.1*) or partially (*Cplx1*, *Hsd17b2*, *Itgb1*, *Kcnk9*, *Mcam*, *Rbm24*, *Tubb6*) restored to normoxic levels by AFL, with only one transcript (*Mir467c*) maintaining expression levels similar to baseline at all timepoints (Fig. 5d). Among these genes, *Mcam* encodes a vascular wall component that plays a prominent role in cancer metastasis and developing tissues by, on the one hand, serving as a receptor for multiple pro-angiogenic factors (as Wnt5a, Netrin-1, FGF4, VEGF-C and Wnt1) and, on the other, allowing rapidly proliferating cells to migrate more effectively via cell–cell and cell-ECM interactions^90^. Importantly, CD146 (encoded by *Mcam*) is a co-receptor required for the phosphorylation of VEGFR-2 upon binding with VEGF-A and the subsequent promotion of EC migration and microvascularization^91^. Thus, its down-regulation by AFL might limit the VEGF-A-induced activation of VEGFR-2 and prevent EC invasion of the vitreous body. Meanwhile, *Itgb1,* which had been briefly discussed earlier, is essential for the formation of stable and mature blood vessels but can also impair sprouting angiogenesis in certain contexts^92^. Hence, its down-regulation by AFL might support a more efficient retinal revascularization by promoting sprouting angiogenesis. Additionally, the RNA binding motif protein 24 (encoded by *Rbm24*) is known to post-transcriptionally regulate the stability of the transcription factor Sox2^93^. As its depletion has been reported to result in anophtalmia or microphtalmia in mice and zebrafish^93^, which are defects similar to those linked to SOX2 deficiency in humans, reduced expression of this gene in OIR retinas might severely impair correct ocular development. In this sense, Complexin (encoded by the *Cplx1* gene) is relevant for the stabilization and timely release of synaptic vesicles^94^ and its modulation by AFL might be related to improvements in synaptic maturation.

Further, *Cd248* is known to be upregulated in response to hypoxia^95^ and to encode a marker of tumour vessel-associated mural cells (Endosialin)^96^ expressed also on newly forming vessels in developing tissues^97^. Therefore, its down-regulation in response to AFL at P17 might be the reflection of reduced levels of hypoxia in the tissue. Interestingly, the expression trends of *Fam19a4* and *Kcna4*, both of which are known to be expressed in RGCs^98,99^, change similarly in normoxic and OIR retinas throughout time. The latter suggests that the normalizing effect of AFL on the expression of these genes at P17 might be linked to a protective effect on such cell population. Meanwhile, the expression dynamics of *Mir1896*, which is upregulated in activated astrocytes and induces the production of inflammatory cytokines and the migration of CD4^+^ T cells in a mouse model of experimental autoimmune encephalitis^100^, suggest that a likely consequence of the hypoxic damage induced to the retina by the OIR protocol might be a period of retinal astrocyte activation.

### A third dose of AFL normalizes the expression of multiple genes whose levels deviate from normoxic ones at P19 in animals that received only two AFL doses

**Figure 6:**
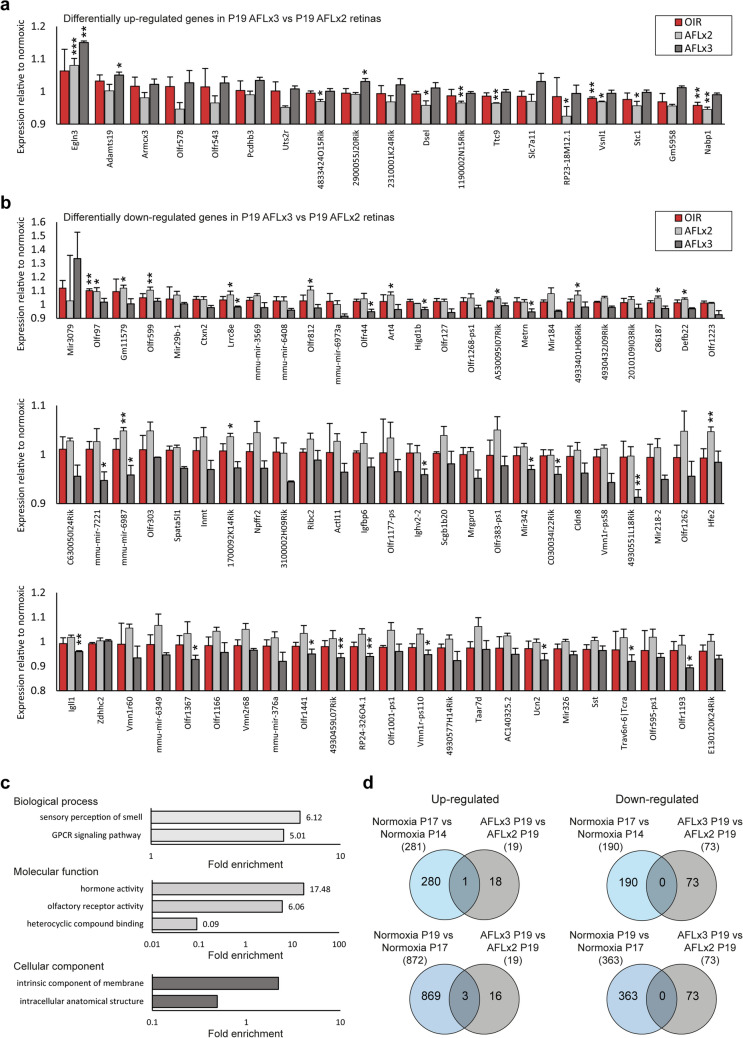
Differentially expressed genes in P19 OIR + AFL(× 3) relative to P19 OIR + AFL(× 2) retinas, GO terms enriched among them, and comparison with differentially regulated genes throughout murine retinal development. (**a,b**) Expression levels of genes differentially up-regulated (**a**) and down-regulated (**b**) in P19 OIR + AFL(× 3) relative to P19 OIR + AFL(× 2) retinas. Expression is shown for P19 OIR, OIR + AFL(× 2) and OIR + AFL(× 3) retinas normalized to the expression level detected in normoxic age-matched tissues. (**c**) GO terms enriched in the list of differentially expressed genes in P19 OIR + AFL(× 3) retinas relative to P19 OIR + AFL(× 2) retinas. Domains: biological process, top; molecular function, middle; cellular component, bottom. (**d**) Venn diagrams of differentially up-regulated (left) and down-regulated (right) genes in P19 OIR + AFL(× 3) retinas relative to P19 OIR + AFL(× 2) ones and during normal retinal development in the P14 to P17 period (top) or in the P17 to P19 timeframe (bottom). In (**a,b**) error bars depict + 1 S.E.M. and statistical significance was calculated relative to expression levels in age-matched normoxic retinas. *P < 0.05; **P < 0.01; ***P < 0.001.

To assess the effect of administering an additional dose of AFL at P17, we compared the transcriptional profiles of P19 animals subjected to the OIR protocol that received two and three AFL injections. This comparison revealed the differential regulation of 92 genes (Supplementary Tables 23–24): 19 up-regulated (Fig. 6a) and 73 down-regulated (Fig. 6b). By comparing the expression levels of these 19 up-regulated genes in OIR, OIR + AFL(× 2) and OIR + AFL(× 3) retinas against their normoxic levels, we determined that while 9 remained dysregulated in OIR + AFL(× 2) tissues, only 3 and 2 were dysregulated in OIR + AFL(× 3) and OIR tissues, respectively (Fig. 6a). Crucially, among these 19 genes, the two that remained dysregulated in OIR at P19, namely *Vsnl1* and *Nabp1*, were also lower in OIR + AFL(× 2) retinas than in normoxic controls and only a third dose of AFL restored their expression. While *Nabp1* has been primarily linked to the DNA damage response and to transcription termination as part of the Integrator complex^101^, enriched expression of *Vsnl1* has been previously detected in rat RGCs using microarrays^102^, which suggests that a third dose of AFL prolongs its protective effects on this cell population. From the clinical perspective, this finding might be of the utmost relevance in light of the very recently reported continuous loss of RGCs in individuals affected by diabetic retinopathy (DR)^103^.

In contrast, from the 73 genes differentially down-regulated in OIR + AFL(× 3) retinas relative to OIR + AFL(× 2) ones, 19 were dysregulated after a third dose of AFL compared to normoxic controls while only 13 were differentially expressed in retinas of animals that received only 2 injections. Nonetheless, a third dose of AFL effectively normalized the expression levels of 11 of these 13 dysregulated genes, including 3 olfactory receptors (*Olfr* genes). Indeed, relative to OIR + AFL(× 2) retinas, 17 *Olfr* genes were down-regulated in OIR + AFL(× 3) tissues, which is in agreement with the identification of “sensory perception of smell” and “olfactory receptor activity” as the most and second most enriched GO terms in the biological process and molecular function domains, respectively (Fig. 6c). In this context, 96 of the 194 G protein-coupled receptors known to be expressed in the retina are putative olfactory receptors^104^. However, given the heterogeneous expression patterns of such receptors in the retina, some being specific to certain cell types and others being ubiquitous^105^, their role in the tissue and the significance of our observations remain to be determined. A third dose of AFL also down-regulated the expression levels of 3 and 13 miRNAs compared to P19 normoxic and OIR + AFL(× 2) retinas, respectively. Considering the prominent role that microRNAs play during retinal development, as well as in pathological conditions^106,107^, and the recent report of certain miRNAs gradually changing their expression levels throughout the progression from non-proliferative to PDR in humans^103^, it is not beyond reason that AFL might partially exert its beneficial effects by modulating the expression level of these transcripts. However, this hypothesis requires future experimental validation.

Finally, we asked whether a third dose of AFL, compared to a two-dose regimen, could promote the occurrence of transcriptional changes characteristic of development. To this aim, we compared the 19 up-regulated and 73 down-regulated genes in P19 OIR + AFL(× 3) retinas relative to P19 OIR + AFL(× 2) samples with those up and down-regulated either from P14 to P17 or from P17 to P19 in the retinas of mice kept in ambient air conditions. Our comparison revealed no overlap in down-regulated genes, with one (*Stc1*) and three (*1190002N15Rik*, *Nabp1*, *Slc7a11*) upregulated genes in P19 OIR + AFL(× 3) retinas found to also increase their expression in normoxia from P14 to P17 and from P17 to P19, respectively (Fig. 6d). In spite of this limited overlap, the upregulation of *Stc1*, which encodes a protein shown to prevent the loss of RGCs in a rat model of optic nerve transection in vivo and in RGC-5 cells damaged with CoCl_2_ in vitro by reducing oxidative damage^108^, and of *Slc7a11*, which encodes an amino acid transport system specific for cysteine and glutamate expressed in RGCs^109^, provides further evidence of the potential neuroprotective properties of AFL.

Moreover, this marginal overlap in differentially expressed genes suggests that AFL does not promote the recapitulation of developmental processes but might instead protect the neuronal retina from hypoxic damage by inhibiting the formation of aberrant microvascular structures and supporting the revascularization of the tissue, in line with our previous publication^22^. In this context, our observations suggest that a regimen with three doses of AFL might result in an enhanced neuroprotective effect on RGCs, a cell population that has been described as susceptible to damage in diabetes and DR^110^.

## Discussion

Ischemic retinopathies as DR and ROP exhibit complex morpho-functional phenotypes. Therapeutic strategies for their treatment focus primarily on inhibiting the emergence and/or worsening of microvascular aberrations, but often fail to directly target the mechanisms giving rise to functional deficits. Moreover, conventional anti-angiogenic therapies targeting solely VEGF-A are limited by the recurrence of neovascularization, a lack of neuronal functional recovery, their inability to regulate inflammation, and the emergence of drug resistance^3,11,13,111^. Here, we investigated the retinal transcriptional response to hypoxia and to AFL administration during the vaso-proliferative and vaso-regenerative phases of the mouse OIR model.

To examine the transcriptional profile of the OIR retina, recent studies have used bulk and single cell RNA-sequencing^24,112^. While these approaches offer the possibility to dissect the heterogeneity of cellular responses with high resolution and to identify novel gene isoforms, they often select for polyadenylated transcripts and might be limited by sequencing depth. In contrast, microarrays robustly detect both highly and lowly expressed genes, non-coding RNAs and predicted sequences^113^. On this basis, microarrays have been extensively used to study the retinal transcriptome^74,113–116^, with us being the first to use them for investigating the response of the OIR retina to AFL administration^22^. Further, as retinopathy gives rise to complex phenotypic features involving and affecting most retinal cell types^3^, studying the tissue as a whole might be advantageous to understand the pathogenesis of retinal ischemic diseases at the systemic level. The latter, however, is at the expense of a reduced capacity to detect subtle changes in transcriptomic profiles within specific cellular populations.

Considering this limitation, we attempted to maximize the sensitivity of our approach by using a low threshold for identifying DRGs (fold-change > 1.2). The latter is in line with a recent study on the human diabetic retina reporting the magnitude of changes in gene expression levels to be below 50%^103^. For stringency, we collected samples for all conditions in triplicate and utilized a confidence interval of 95% for all comparisons. To validate our results, we aimed at comparing them with previously published datasets. However, most studies used distinct time-points or focused on just a subset of genes^113^. Moreover, when comparing with suitable studies, we detected divergences potentially arising from thresholding differences, the presence of sex chromosome-linked genes (omitted in our study), the use of distinct animal species, and/or modifications to the OIR protocol^19^ (we started hyperoxia one day earlier). For instance, of 662 DRGs we identified at P17 in OIR retinas, only 80 overlapped with those found by Guarischi-Sousa and colleagues at the same time-point^112^ (153 in total). In a different study, 43 and 1622 DRGs were identified in OIR retinas at P13 and P18, respectively^74^; in contrast, we detected 272 and 398 DRGs at P14 and P19. Notwithstanding, the processes regulated by the genes we identified are consistent with those reported in other studies on the OIR retina^114,117^, including sprouting angiogenesis, metabolism and inflammation.

Presently, the mechanisms mediating neovascular tuft clearance remain understudied. In a recent report, leukocytes were described as central players in this process^24^. In agreement, a number of GO terms detected as enriched in our study were related to leukocyte migration and chemotaxis. Moreover, the proteins encoded by the genes associated to these terms included important regulators of leukocyte adhesion and transendothelial migration, as well as modulators of monocyte infiltration and neutrophil migration. Additionally, as high VEGF-A levels have been suggested to lead to leukostasis in the eye^118^, its neutralization by AFL might expedite leukocyte recruitment to neovascular tufts for their subsequent clearance. In this context, our findings are in line with previous reports and hint at the potential regulatory role of AFL on this process.

The number of DRGs detected in response to two doses of AFL at P17 (149) was similar to that observed at P19 after three doses (151). Interestingly, however, no single gene was differentially regulated in all treatment regimens. Yet, the most relevant target of AFL identified in our study might be *Angpt2*, as its expression was consistently elevated at all examined time points in OIR retinas, in agreement with a previous report^119^, and downregulated by AFL at P14, P17 and P19 after 2 doses; in P19(× 3) retinas its fold-change was -1.16 (*P* value = 0.09). Ang2 is an antagonist of the tyrosine kinase receptor Tie2 whose importance in vascular development is difficult to overstate. While the expression of Ang2 is responsive to hypoxia^120^ and its dysregulation is characteristic of disease^121^, its blockade has been shown to reduce tumour vascularization and to inhibit metastasis in mammary gland carcinomas and pancreatic insulinomas^122^. Additionally, Ang2 has been implicated in OIR^123^ and described to be up-regulated in the serum of type 2 diabetic patients with either non-proliferative DR or PDR compared to patients without retinopathy^124^. These findings have attracted interest in the possibility of pharmacologically regulating the activity of this agent, with clinical trials assessing the efficacy of neutralizing this molecule currently ongoing^125^. In this sense, the bispecific anti-VEGF/anti-Ang2 antibody Faricimab has been shown to be more effective than sole anti-VEGF therapy in an animal model of laser-induced choroidal neovascularization^126^ and studies evaluating the therapeutic efficacy of simultaneously blocking VEGF-A and Ang2 are ongoing^127^. As *Angpt2* expression is increased in response to stabilization of the hypoxia-inducible factor^2^, our results suggest that AFL is effective in reducing retinal hypoxia.

Two more genes prominently involved in angiogenesis and modulated by AFL were *Esm1* and *Cd34*. *Esm1* has been previously reported as a top overexpressed gene in OIR retinas at P17^113,119^ and plays a role in both pathological and physiological angiogenesis, modulating VEGF bioavailability and regulating leukocyte extravasation^128^. Moreover, increased expression of *Esm1* enhances the proliferative capacity, colony forming ability, migration and invasiveness of radiotherapy-resistant breast cancer cells all by itself, with its down-regulation reversing these effects^129^. Meanwhile, Cd34 has been previously shown to promote pathological neovascularization in the OIR retina^130^. Therefore, the downregulation of these genes might constitute a relevant mechanism by which AFL inhibits the formation of neovascular tufts while promoting their clearance and permitting the regeneration of the retinal microvascular network. Together, our observations suggest that the capacity of AFL to concomitantly modulate the synthesis and deposition of ECM components, which regulate adhesion, together with its effects on *Angpt2* levels, might represent key mechanisms by which it orchestrates a more efficient recovery of the retinal microvasculature. Specifically, we hypothesize that lower levels of Ang2 are central to supress the exuberant vasoproliferation characteristic of the OIR retina while the normalization in the composition of the basement membrane might allow ECs to migrate and assemble functional vessels that contribute to reducing tissue hypoxia more effectively. Moreover, besides genes, we reveal that AFL exerts regulatory effects over certain miRNAs. Given the broad spectrum of binding partners of each individual miRNA species, their ease of synthesis and their limited toxicity, these small RNAs might be valuable not just as disease biomarkers but also as powerful therapeutic agents and targets.

Beyond its marked regulatory effects on angiogenesis, AFL also modulated inflammatory processes and exhibited neuroprotective features, as revealed by the enrichment of GO terms associated to wound healing and synaptic transmission. Moreover, considering that ion flux into photoreceptors is a critical process for vision often disrupted in diabetes^3^, the enrichment of GO terms associated to potassium, calcium, and chloride ion transport in response to AFL administration provides further evidence of its beneficial effects on visual perception, in line with our previous findings^22^. The latter is of key relevance as damage to retinal neurons can be detected before vascular abnormalities in human patients with diabetes^127^. Modulating not only angiogenesis but also a broader spectrum of processes relevant to retinal function and apparently protecting not only dopaminergic amacrine cells from hypoxic damage, as we have previously reported^22^, but potentially also RGCs, AFL might be a more powerful agent than previously recognized. In this sense, our exploratory study might serve as a valuable resource for informing future research aimed at the identification of additional molecular targets whose pharmacological regulation might be beneficial for the treatment of multiple retinopathies, including diabetic retinopathy and retinopathy of prematurity.

## Methods

### Study approval

Animal experiments were performed in strict accordance with German Animal Welfare legislation and adhering to the ARRIVE guidelines. All experimental protocols (TVV26/2017) were reviewed and approved by the Landesdirektion Dresden with the approval number DD24.1‐5131/394/29. All efforts were made to minimize animal suffering. Anesthesia overdosing prior to eye collection was verified by the absence of interdigital withdrawal reflexes. Pharmacological euthanasia was followed by decapitation. All mice were acquired from Charles River Laboratories (Sulzfeld, Germany), either to breed in‐house or as pregnant females. Newborns were allocated to experimental groups arbitrarily and irrespective of their sex.

### OIR protocol and Aflibercept administration

Retinal neovascularization was induced by the OIR protocol^19^. The protocol consisted in exposing C57BL/6J (IMSR Cat# JAX:000664, RRID:IMSR_JAX:000664) mouse pups and their mothers to 75% oxygen from P6 to P11 (P0 = birthdate); before and afterwards, all mice were housed in ambient air conditions in cages with wood shavings beddings and using a 12 h light/dark cycle. Temperature control and water quality was continuously monitored and followed the regulations of the TU Dresden Medical Faculty Animal House. Aflibercept (Bayer Vital GmbH, Leverkusen, Germany) diluted in sterile PBS (Sigma‐Aldrich, Munich, Germany) was administered intra‐peritoneally to half the pups in each litter at 25 mg/kg BW on one of two regimens: either on P13 and P15 or on P13, P15 and P17. Non-injected OIR littermates were kept as controls. Half the animals in each litter were arbitrarily allocated by the experimenter to the Aflibercept‐treated group and the remaining half to the non‐injected OIR control group. No exclusion criteria were enforced and all animals were considered. In total, 12 mice were used in the present study. Non-OIR animals (normoxic controls) were reared in ambient air conditions. Normoxic controls were not injected with any substance before being sacrificed. All mice were sacrificed between 13:00 and 17:00 h (CET + 1) by an overdose of ketamine (300 mg/kg BW; Ratiopharm, Ulm, Germany) and xylazine (30 mg/kg BW; Ratiopharm). The effectiveness of the OIR protocol was ascertained by confirming the presence of neovascularization and vaso-obliteration by immunofluorescence in flat-mounts of retinas from animals used for a different study which were inside the hyperoxia chamber at the same time-points as the animals used for this study. The oxygen concentration in the hyperoxia chamber was monitored daily to confirm deviations did not exceed ± 2%, which from previous experience is considered an acceptable range.

### RNA isolation

Three P19 mice per condition (normoxia, OIR, OIR + AFLx2, OIR + AFLx3) were used for RNA isolation. The retinas of each mouse were dissected in PBS at 37 °C and subsequently pooled together. Warm PBS was replaced by 750 µL TRIzol (Thermo Fisher Scientific), and retinal tissues were disrupted by 10 rounds of aspiration/ejection through 18G needles and 10 more rounds through 23G needles. After 5 min incubation at room temperature, 150 µL chloroform (Merck, Darmstadt, Hesse, Germany) were added and samples were vortexed, incubated an additional 15 min at RT, and centrifuged 20 min at 12,000*g* and 4 °C. The aqueous phase was subsequently collected and mixed with 1.5 µl RNA‐grade glycogen (Thermo Fisher Scientific) and 375 µl isopropanol (Merck). Samples were subsequently vortexed and centrifuged 20 min at 12,000*g* and 4 °C. Pellets were washed twice in 1 ml 75% ethanol, air‐dried 10–20 min, and re‐suspended in 20 µL RNAse‐free water. Aliquots of 1 µL RNA per sample were kept separate for quality control measurements avoiding unnecessary freeze–thaw cycles.

### Gene expression profiling, GO Term and KEGG pathway enrichment analysis

Isolated RNA was sent to the Gene Expression Facility of the Max Planck Institute for Cell Biology and Genetics (Dresden, Saxony, Germany). There, biotinylated cDNA samples were prepared according to the GeneChip WT PLUS Reagent Kit (Cat. Nr. 902280) protocol using 250 ng total RNA as starting material. 5.5 ug of biotinylated and fragmented single-stranded cDNA were hybridized for 16 h at 45 °C on Affymetrix GeneChip Mouse Whole Transcriptome Arrays according to the GeneChip® WT PLUS Reagent Kit User Manual. GeneChips were washed and stained in the Affymetrix Fluidics Station 400. GeneChips were subsequently scanned using the Hewlett-Packard GeneArray Scanner G2500A. Scanned images were processed with the Transcriptome Analysis Console (TAC) Software (Affymetrix) using default parameters to obtain background subtracted and summarized probe sets. Data (.CEL files) generated in these microarray chips was imported into GeneSpring v14.9 (Agilent Technologies) together with the .CEL files generated in our previous study (GEO accession number: GSE124956) describing the response of the OIR retina to AFL administration at earlier time-points, namely P14 and P17. By normalizing all datasets together using the Robust Multi-array Average (RMA) approach, we aimed at ensuring comparability across all datasets. No baseline transformation was conducted. Normalized expression levels were used to identify differentially regulated genes in GeneSpring. A gene was considered to be differentially expressed if it exhibited a fold-change above or below 1.2 and a p-value below 0.05 as a result of pairwise comparison (unpaired *t* test) across time-points or conditions. False discovery rate (FDR) was not used. Transcripts expressed from the X or Y chromosome were not considered for analysis. Data were exported as .csv files for further analysis. The microarray data produced in this study has been deposited in the NCBI Gene Expression Omnibus database (accession number GSE137705). Venn diagrams were created using InteractiVenn^131^ and refined in Adobe Illustrator. Lists of differentially regulated genes were used as input in PANTHER^25–27^ and ShinyGO^132^ for identifying enriched GO terms and KEGG Pathways, respectively. PANTHER settings were used as default, i.e. using a Fisher’s Exact test and a False Discovery Rate correction. When a high number of terms or pathways were detected, a subset of the most specific terms was arbitrarily selected and used for depiction in figures.

## Supplementary Information

Supplementary Figures.

Supplementary Table 1.

Supplementary Table 2.

Supplementary Table 3.

Supplementary Table 4.

Supplementary Table 5.

Supplementary Table 6.

Supplementary Table 7.

Supplementary Table 8.

Supplementary Table 9.

Supplementary Table 10.

Supplementary Table 11.

Supplementary Table 12.

Supplementary Table 13.

Supplementary Table 14.

Supplementary Table 15.

Supplementary Table 16.

Supplementary Table 17.

Supplementary Table 18.

Supplementary Table 19.

Supplementary Table 20.

Supplementary Table 21.

Supplementary Table 22.

Supplementary Table 23.

Supplementary Table 24.

## Data Availability

The microarray data produced in this study, including raw files (.CEL) and an expression matrix with absolute and normalized intensity values for each entity detected, have been deposited in the NCBI Gene Expression Omnibus database (accession number GSE137705). All data from our previous study describing the response of the OIR retina to AFL administration at earlier time-points, namely P14 and P17, has also been deposited in the NCBI Gene Expression Omnibus and is publicly available (GEO accession number: GSE124956). Differential gene expression data generated for this research article is included as Supplementary Information.

## References

[1] Hansen RM, Moskowitz A, Akula JD, Fulton AB (2017). The neural retina in retinopathy of prematurity. Prog. Retin. Eye Res..

[2] Campochiaro PA, Akhlaq A (2020). Sustained suppression of VEGF for treatment of retinal/choroidal vascular diseases. Prog. Retin. Eye Res..

[3] Tonade D, Kern TS (2020). Photoreceptor cells and RPE contribute to the development of diabetic retinopathy. Prog. Retin. Eye Res..

[4] Stitt AW (2016). The progress in understanding and treatment of diabetic retinopathy. Prog. Retin. Eye Res..

[5] Shweiki D, Itin A, Soffer D, Keshet E (1992). Vascular endotehlial growth factor induced by hypoxia may mediate hypoxia-induced angiogenesis. Nature.

[6] Witmer AN, Vrensen GFJM, Van Noorden CJF, Schlingemann RO (2003). Vascular endothelial growth factors and angiogenesis in eye disease. Prog. Retin. Eye Res..

[7] Alon T (1995). Vascular endothelial growth factor acts as a survival factor for newly formed retinal vessels and has implications for retinopathy of prematurity. Nat. Med..

[8] Jain RK (2007). Angiogenesis in brain tumours. Nat. Rev. Neurosci..

[9] Kim KJ (1984). Inhibition of vascular endothelial growth factor-induced angiogenesis suppresses tumour growth in vivo. Nature.

[10] Gasparini G, Longo R, Toi M, Ferrara N (2005). Angiogenic inhibitors: A new therapeutic strategy in oncology. Nat. Clin. Pract. Oncol..

[11] Aiello LP (1995). Suppression of retinal neovascularization in vivo by inhibition of vascular endothelial growth factor (VEGF) using soluble VEGF-receptor chimeric proteins. Proc. Natl. Acad. Sci. USA.

[12] Adamis AP (1996). Inhibition of vascular endothelial growth factor prevents retinal ischemia—Associated iris neovascularization in a nonhuman primate. Arch. Ophthalmol..

[13] Sene A, Chin-Yee D, Apte RS (2015). Seeing through VEGF: Innate and adaptive immunity in pathological angiogenesis in the eye. Trends Mol. Med..

[14] Carmeliet P (2001). Synergism between vascular endothelial growth factor and placental growth factor contributes to angiogenesis and plasma extravasation in pathological conditions. Nat. Med..

[15] Jászai J, Schmidt MHH (2019). Trends and Challenges in Tumor Anti-Angiogenic Therapies. Cells.

[16] Stewart MW, Grippon S, Kirkpatrick P (2012). Fresh from the pipeline: Aflibercept. Nat. Rev. Drug Discov..

[17] Holash J (2002). VEGF-Trap: A VEGF blocker with potent antitumor effects. Proc. Natl. Acad. Sci. USA.

[18] Simons M, Gordon E, Claesson-Welsh L (2016). Mechanisms and regulation of endothelial VEGF receptor signalling. Nat. Rev. Mol. Cell Biol..

[19] Smith LEH (1994). Oxygen-induced retinopathy in the mouse. Investig. Ophthalmol. Vis. Sci..

[20] Stahl A (2010). The mouse retina as an angiogenesis model. Investig. Ophthalmol. Vis. Sci..

[21] Scott A, Fruttiger M (2010). Oxygen-induced retinopathy: A model for vascular pathology in the retina. Eye.

[22] Rojo Arias JE (2020). VEGF-Trap is a potent modulator of vasoregenerative responses and protects dopaminergic amacrine network integrity in degenerative ischemic neovascular retinopathy. J. Neurochem..

[23] Korn C, Augustin HG (2015). Mechanisms of vessel pruning and regression. Dev. Cell.

[24] Binet F (2020). Neutrophil extracellular traps target senescent vasculature for tissue remodeling in retinopathy. Science (80-).

[25] Ashburner M (2000). Gene ontology: Tool for the unification of biology The Gene Ontology Consortium. Nat. Genet..

[26] Carbon S (2019). The Gene Ontology Resource: 20 years and still GOing strong. Nucleic Acids Res..

[27] Mi H, Muruganujan A, Ebert D, Huang X, Thomas PD (2019). PANTHER version 14: More genomes, a new PANTHER GO-slim and improvements in enrichment analysis tools. Nucleic Acids Res..

[28] Caprara C, Grimm C (2012). From oxygen to erythropoietin: Relevance of hypoxia for retinal development, health and disease. Prog. Retin. Eye Res..

[29] Sperandio S (2009). The transcription factor Egr1 regulates the HIF-1α gene during hypoxia. Mol. Carcinog..

[30] Marchand M, Monnot C, Muller L, Germain S (2019). Extracellular matrix scaffolding in angiogenesis and capillary homeostasis. Semin. Cell Dev. Biol..

[31] Pöschl E (2004). Collagen IV is essential for basement membrane stability but dispensable for initiation of its assembly during early development. Development.

[32] Zhao SH (2009). Vascular endothelial growth factor upregulates expression of annexin A2 in vitro and in a mouse model of ischemic retinopathy. Mol. Vis..

[33] Whitehead M, Osborne A, Widdowson PS, Yu-Wai-Man P, Martin KR (2019). Angiopoietins in diabetic retinopathy: Current understanding and therapeutic potential. J. Diabetes Res..

[34] Kotaka M (2000). Interaction of hCLIM1, an enigma family protein, with α-actinin 2. J. Cell. Biochem..

[35] Laboissonniere LA (2019). Molecular signatures of retinal ganglion cells revealed through single cell profiling. Sci. Rep..

[36] Van Breevoort D (2012). Proteomic screen identifies IGFBP7 as a novel component of endothelial cell-specific Weibel-Palade bodies. J. Proteome Res..

[37] Komiya E (2014). Angiomodulin, a marker of cancer vasculature, is upregulated by vascular endothelial growth factor and increases vascular permeability as a ligand of integrin avb3. Cancer Med..

[38] Hooper AT (2009). Angiomodulin is a specific marker of vasculature and factor-A – Dependent neoangiogenesis. Circ. Res..

[39] Lofqvist C (2009). Quantification and localization of the IGF/insulin system expression in retinal blood vessels and neurons during oxygen-induced retinopathy in mice. Investig. Ophthalmol. Vis. Sci..

[40] Deliyanti D (2017). Foxp3+ Tregs are recruited to the retina to repair pathological angiogenesis. Nat. Commun..

[41] Muller WA, Weigl SA, Deng X, Phillips DM (1993). PECAM-1 is required for transendothelial migration of leukocytes. J. Exp. Med..

[42] Verzola D (2017). Myostatin mediates abdominal aortic atherosclerosis progression by inducing vascular smooth muscle cell dysfunction and monocyte recruitment. Sci. Rep..

[43] Guo Q (2019). Integrin β1-enriched extracellular vesicles mediate monocyte adhesion and promote liver inflammation in murine NASH. J. Hepatol..

[44] Morote-Garcia JC, Napiwotzky D, Köhler D, Rosenberger P (2012). Endothelial Semaphorin 7A promotes neutrophil migration during hypoxia. Proc. Natl. Acad. Sci. USA.

[45] Hu S (2018). Vascular Semaphorin 7A upregulation by disturbed flow promotes atherosclerosis through endothelial β1 integrin. Arterioscler. Thromb. Vasc. Biol..

[46] Klose A, Zigrino P, Mauch C (2013). Monocyte/macrophage MMP-14 modulates cell infiltration and T-cell attraction in contact dermatitis but not in murine wound healing. Am. J. Pathol..

[47] De Jager SCA (2013). Leukocyte-specific CCL3 deficiency inhibits atherosclerotic lesion development by affecting neutrophil accumulation. Arterioscler. Thromb. Vasc. Biol..

[48] Castro CN (2020). NCKAP1L defects lead to a novel syndrome combining immunodeficiency, lymphoproliferation, and hyperinflammation. J. Exp. Med..

[49] Boeck M (2020). Temporospatial distribution and transcriptional profile of retinal microglia in the oxygen-induced retinopathy mouse model. Glia.

[50] Beli E (2016). CX3CR1 deficiency accelerates the development of retinopathy in a rodent model of type 1 diabetes. J. Mol. Med. (Berl).

[51] Ronning KE, Karlen SJ, Miller EB, Burns ME (2019). Molecular profiling of resident and infiltrating mononuclear phagocytes during rapid adult retinal degeneration using single-cell RNA sequencing. Sci. Rep..

[52] Fischer F, Martin G, Agostini HT (2011). Activation of retinal microglia rather than microglial cell density correlates with retinal neovascularization in the mouse model of oxygen-induced retinopathy. J. Neuroinflammation.

[53] Dejda A (2014). Neuropilin-1 mediates myeloid cell chemoattraction and influences retinal neuroimmune crosstalk. J. Clin. Investig..

[54] Wang W (2015). FAM19A4 is a novel cytokine ligand of formyl peptide receptor 1 (FPR1) and is able to promote the migration and phagocytosis of macrophages. Cell. Mol. Immunol..

[55] Scurr I (2011). Cantú syndrome: Report of nine new cases and expansion of the clinical phenotype. Am. J. Med. Genet. Part A.

[56] Yang D, Zhang X, Hughes BA (2008). Expression of inwardly rectifying potassium channel subunits in native human retinal pigment epithelium. Exp. Eye Res..

[57] Joshi S, Wei J, Bishopric NH (2016). A cardiac myocyte-restricted Lin28/let-7 regulatory axis promotes hypoxia-mediated apoptosis by inducing the AKT signaling suppressor PIK3IP1. Biochim. Biophys. Acta Mol. Basis Dis..

[58] Zhang Y (2015). Hypoxia-induced proliferation in mesenchymal stem cells and angiotensin II-mediated PI3K/AKT pathway. Cell Biochem. Funct..

[59] Skiles WM (2018). Oxygen-induced alterations in the expression of chromatin modifying enzymes and the transcriptional regulation of imprinted genes. Gene Expr. Patterns.

[60] Furuhata T, Tokino T, Urano T, Nakamura Y (1996). Isolation of a novel GPI-anchored gene specifically regulated by p53; Correlation between its expression and anti-cancer drug sensitivity. Oncogene.

[61] Balasubramanian S (2009). Comparative analysis of processed ribosomal protein pseudogenes in four mammalian genomes. Genome Biol..

[62] Matsson H (2004). Targeted disruption of the ribosomal protein S19 gene is lethal prior to implantation. Mol. Cell. Biol..

[63] Uechi T (2006). Ribosomal protein gene knockdown causes developmental defects in zebrafish. PLoS ONE.

[64] Filip AM (2009). Ribosomal protein S19 interacts with macrophage migration inhibitory factor and attenuates its pro-inflammatory function. J. Biol. Chem..

[65] Wang M (2018). LncRNA NKILA upregulation mediates oxygen glucose deprivation/re-oxygenation-induced neuronal cell death by inhibiting NF-κB signaling. Biochem. Biophys. Res. Commun..

[66] Zhou Q, Zhou L, Qian J, Yuan Z, Chen Z (2018). NKILA inhibition protects retinal pigment epithelium cells from hypoxia by facilitating NFκB activation. Biochem. Biophys. Res. Commun..

[67] Adijanto J (2014). The retinal pigment epithelium utilizes fatty acids for ketogenesis implications for metabolic coupling with the outer retina. J. Biol. Chem..

[68] Ristic B, Bhutia YD, Ganapathy V (2017). Cell-surface G-protein-coupled receptors for tumor-associated metabolites: A direct link to mitochondrial dysfunction in cancer. Biochim. Biophys. Acta Rev. Cancer.

[69] Rohlenova K (2020). Single-cell RNA sequencing maps endothelial metabolic plasticity in pathological angiogenesis. Cell Metab..

[70] Roshanbin S (2016). Histological characterization of orphan transporter MCT14 (SLC16A14) shows abundant expression in mouse CNS and kidney. BMC Neurosci..

[71] Zhang Y (2014). An RNA-sequencing transcriptome and splicing database of glia, neurons, and vascular cells of the cerebral cortex. J. Neurosci..

[72] Fisel P, Schaeffeler E, Schwab M (2018). Clinical and functional relevance of the monocarboxylate transporter family in disease pathophysiology and drug therapy. Clin. Transl. Sci..

[73] Suply T (2017). A natural ligand for the orphan receptor GPR15 modulates lymphocyte recruitment to epithelia. Sci. Signal..

[74] Recchia FM, Xu L, Penn JS, Boone B, Dexheimer PJ (2010). Identification of genes and pathways involved in retinal neovascularization by microarray analysis of two animal models of retinal angiogenesis. Investig. Ophthalmol. Vis. Sci..

[75] Harakalova M (2012). Dominant missense mutations in ABCC9 cause Cantúsyndrome. Nat. Genet..

[76] York NW (2020). Kir61- and SUR2-dependent KATP over-activity disrupts intestinal motility in murine models of Cantu Syndrome. JCI Insight.

[77] Ettaiche M (2001). ATP-sensitive potassium channels (KATP) in retina: A key role for delayed ischemic tolerance. Brain Res..

[78] DiMaio TA (2008). Attenuation of retinal vascular development and neovascularization in PECAM-1-deficient mice. Dev. Biol..

[79] Glise L (2019). Disturbed laminar blood flow causes impaired fibrinolysis and endothelial fibrin deposition in vivo. Thromb. Haemost..

[80] Lin CY (2017). ADAM9 promotes lung cancer progression through vascular remodeling by VEGFA, ANGPT2, and PLAT. Sci. Rep..

[81] Bence M, Kereszturi E, Mozes V, Sasvari-Szekely M, Keszler G (2009). Hypoxia-induced transcription of dopamine D3 and D4 receptors in human neuroblastoma and astrocytoma cells. BMC Neurosci..

[82] Klitten LL (2009). Localization and regulation of dopamine receptor D4 expression in the adult and developing rat retina. Exp. Eye Res..

[83] Okumichi H, Mizukami M, Kiuchi Y, Kanamoto T (2008). GABAA receptors are associated with retinal ganglion cell death induced by oxidative stress. Exp. Eye Res..

[84] Grünert U (2000). Distribution of GABA and glycine receptors on bipolar and ganglion cells in the mammalian retina. Microsc. Res. Tech..

[85] Wässle H (2004). Parallel processing in the mammalian retina. Nat. Rev. Neurosci..

[86] Ishii T (2017). Novel channel-mediated choline transport in cholinergic neurons of the mouse retina. J. Neurophysiol..

[87] Han HJ, Tokino T, Nakamura Y (1998). CSR, a scavenger receptor-like protein with a protective role against cellular damage caused by UV irradiation and oxidative stress. Hum. Mol. Genet..

[88] Liu Y (2009). Identification of small Sca-1+, Lin-, CD45- multipotential cells in the neonatal murine retina. Exp. Hematol..

[89] Shao Z (2018). Young bone marrow Sca-1 cells protect aged retina from ischaemia-reperfusion injury through activation of FGF2. J. Cell. Mol. Med..

[90] Wang Z (2020). CD146, from a melanoma cell adhesion molecule to a signaling receptor. Signal Transduct. Target. Ther..

[91] Jiang T (2012). CD146 is a coreceptor for VEGFR-2 in tumor angiogenesis. Blood.

[92] Yamamoto H (2015). Integrin β1 controls VE-cadherin localization and blood vessel stability. Nat. Commun..

[93] Dash S (2020). The master transcription factor SOX2, mutated in anophthalmia/microphthalmia, is post-transcriptionally regulated by the conserved RNA-binding protein RBM24 in vertebrate eye development. Hum. Mol. Genet..

[94] Chang S (2015). Complexin stabilizes newly primed synaptic vesicles and prevents their premature fusion at the mouse calyx of held synapse. J. Neurosci..

[95] Ohradanova A (2008). Hypoxia upregulates expression of human endosialin gene via hypoxia-inducible factor 2. Br. J. Cancer.

[96] Christian S (2008). Endosialin (Tem1) is a marker of tumor-associated myofibroblasts and tumor vessel-associated mural cells. Am. J. Pathol..

[97] Simonavicius N (2012). Pericytes promote selective vessel regression to regulate vascular patterning. Blood.

[98] Tran NM (2019). Single-cell profiles of retinal ganglion cells differing in resilience to injury reveal neuroprotective genes. Neuron.

[99] Höltje M (2007). Differential distribution of voltage-gated potassium channels Kv 1.1-Kv1.6 in the rat retina during development. J. Neurosci. Res..

[100] Liu X (2019). MiR-409-3p and MiR-1896 co-operatively participate in IL-17-induced inflammatory cytokine production in astrocytes and pathogenesis of EAE mice via targeting SOCS3/STAT3 signaling. Glia.

[101] Skaar JR (2015). The Integrator complex controls the termination of transcription at diverse classes of gene targets. Cell Res..

[102] Ivanov D, Dvoriantchikova G, Nathanson L, McKinnon SJ, Shestopalov VI (2006). Microarray analysis of gene expression in adult retinal ganglion cells. FEBS Lett..

[103] Becker K (2021). In-depth transcriptomic analysis of human retina reveals molecular mechanisms underlying diabetic retinopathy. Sci. Rep..

[104] Pronin A (2014). Expression of olfactory signaling genes in the eye. PLoS ONE.

[105] Jovancevic N (2017). Deep sequencing of the human retinae reveals the expression of odorant receptors. Front. Cell. Neurosci..

[106] Zuzic M, Arias JER, Wohl SG, Busskamp V (2019). Retinal miRNA functions in health and disease. Genes (Basel).

[107] Pawlick JS, Zuzic M, Pasquini G, Swiersy A, Busskamp V (2021). MiRNA regulatory functions in photoreceptors. Front. Cell Dev. Biol..

[108] Kim SJ (2013). Stanniocalcin-1 protects retinal ganglion cells by inhibiting apoptosis and oxidative damage. PLoS ONE.

[109] Dun Y (2006). Expression of the cystine-glutamate exchanger (xc-) in retinal ganglion cells and regulation by nitric oxide and oxidative stress. Cell Tissue Res..

[110] Ng DSK (2016). Retinal ganglion cell neuronal damage in diabetes and diabetic retinopathy. Clin. Exp. Ophthalmol..

[111] Bergers G, Hanahan D (2008). Modes of resistance to anti-angiogenic therapy. Nat. Rev. Cancer.

[112] Guarischi-Sousa R (2019). A transcriptome-based signature of pathological angiogenesis predicts breast cancer patient survival. PLOS Genet..

[113] Zasada M (2019). Short- and long-term impact of hyperoxia on the blood and retinal cells’ transcriptome in a mouse model of oxygen-induced retinopathy. Pediatr. Res..

[114] Ishikawa K, Yoshida S, Kadota K, Nakamura T, Niiro H (2010). Gene expression profile of hyperoxic and hypoxic retinas in a mouse model of oxygen-induced retinopathy. Investig. Ophthalmol. Vis. Sci..

[115] Zasada M (2020). Transcriptome analysis reveals dysregulation of genes involved in oxidative phosphorylation in a murine model of retinopathy of prematurity. Pediatr. Res..

[116] Natoli R, Provis J, Valter K, Stone J (2008). Gene regulation induced in the C57BL/6J mouse retina by hyperoxia: A temporal microarray study. Mol. Vis..

[117] Sato T (2009). Comprehensive gene-expression profile in murine oxygen-induced retinopathy. Br. J. Ophthalmol..

[118] Liu, Y. *et al.* Reversible retinal vessel closure from VEGF-induced leukocyte plugging. *JCI Insight* **2**, e95530 (2017). 10.1172/jci.insight.95530 PMC5621911 28931763

[119] Calzi, S. L. *et al.* Progenitor cell combination normalizes retinal vascular development in the oxygen-induced retinopathy (OIR) model. *JCI Insight* **4**, e129224 (2019). 10.1172/jci.insight.129224 PMC6948778 31672944

[120] Kelly BD (2003). Cell type-specific regulation of angiogenic growth factor gene expression and induction of angiogenesis in nonischemic tissue by a constitutively active form of hypoxia-inducible factor 1. Circ. Res..

[121] Akwii RG, Sajib MS, Zahra FT, Mikelis CM (2019). Role of angiopoietin-2 in vascular physiology and pathophysiology. Cells.

[122] Mazzieri R (2011). Targeting the ANG2/TIE2 axis inhibits tumor growth and metastasis by impairing angiogenesis and disabling rebounds of proangiogenic myeloid cells. Cancer Cell.

[123] Hackett SF, Wiegand S, Yancopoulos G, Campochiaro PA (2002). Angiopoietin-2 plays an important role in retinal angiogenesis. J. Cell. Physiol..

[124] Khalaf N (2017). Role of angiopoietins and Tie-2 in diabetic retinopathy. Electron. Physician.

[125] Leong A, Kim M (2020). The angiopoietin-2 and tie pathway as a therapeutic target for enhancing antiangiogenic therapy and immunotherapy in patients with advanced cancer. Int. J. Mol. Sci..

[126] Regula JT (2019). Targeting key angiogenic pathways with a bispecific Cross MA b optimized for neovascular eye diseases. EMBO Mol. Med..

[127] Apte RS, Chen DS, Ferrara N (2019). Review VEGF in signaling and disease: Beyond discovery and development. Cell.

[128] Rocha SF (2014). Esm1 modulates endothelial tip cell behavior and vascular permeability by enhancing VEGF bioavailability. Circ. Res..

[129] Jin H (2020). Esm-1 overexpression is involved in increased tumorigenesis of radiotherapy-resistant breast cancer cells. Cancers (Basel).

[130] Siemerink MJ (2016). CD34 promotes pathological epi-retinal neovascularization in a mouse model of oxygen-induced retinopathy. PLoS ONE.

[131] Heberle H, Meirelles VG, da Silva FR, Telles GP, Minghim R (2015). InteractiVenn: A web-based tool for the analysis of sets through Venn diagrams. BMC Bioinform..

[132] Ge SX, Jung D, Jung D, Yao R (2020). ShinyGO: A graphical gene-set enrichment tool for animals and plants. Bioinformatics.

