# Supplementary material for: Gene expression profile of the murine ischemic retina and its response to Aflibercept (VEGF-Trap)
Source: Sci Rep. 2021 Jul 28;11:15313. doi: 10.1038/s41598-021-94500-1 (PMC8319207; doi:10.1038/s41598-021-94500-1)
Supplement: Supplementary file 1 — Supplementary Figures. [file 41598_2021_94500_MOESM1_ESM.docx]

**SUPPLEMENTARY MATERIAL**

**Gene expression profile of the murine ischemic retina and its response to Aflibercept (VEGF-Trap)**

Jesús Eduardo **Rojo Arias**^1, 2, *^**,** József **Jászai**^1, *^

^1^ Department of Anatomy, Medical Faculty Carl Gustav Carus, Technische Universität Dresden, Saxony, Germany

^2^ Present address: Wellcome-MRC Cambridge Stem Cell Institute, Jeffrey Cheah Biomedical Centre, Cambridge Biomedical Campus, University of Cambridge, Cambridge, UK

*Correspondence:

Jesús Eduardo Rojo Arias: jer72@cam.ac.uk

József Jászai: jozsef.jaszai@tu-dresden.de

*
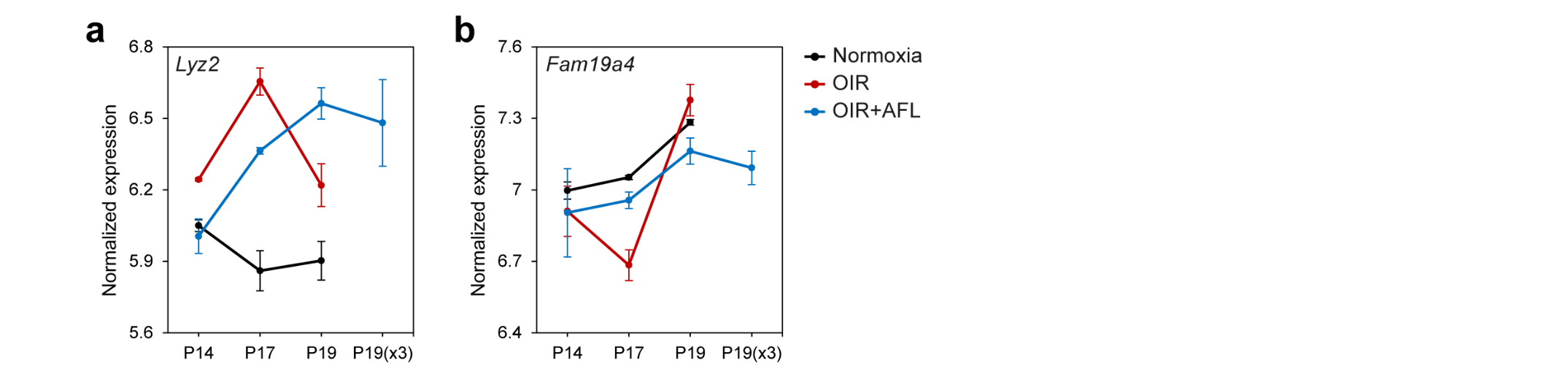
*

**Supplementary Figure 1.** Normalized expression levels of the *Lyz2* (**a**) and *Fam19a4* (**b**) genes at each timepoint (P14, P17, P19) and condition (Normoxia, OIR, OIR+AFL) examined. The additional P19(x3) time-point refers to retinas of OIR animals that received 3 doses of AFL and were examined at P19. Error bars depict ±1 S.E.M.


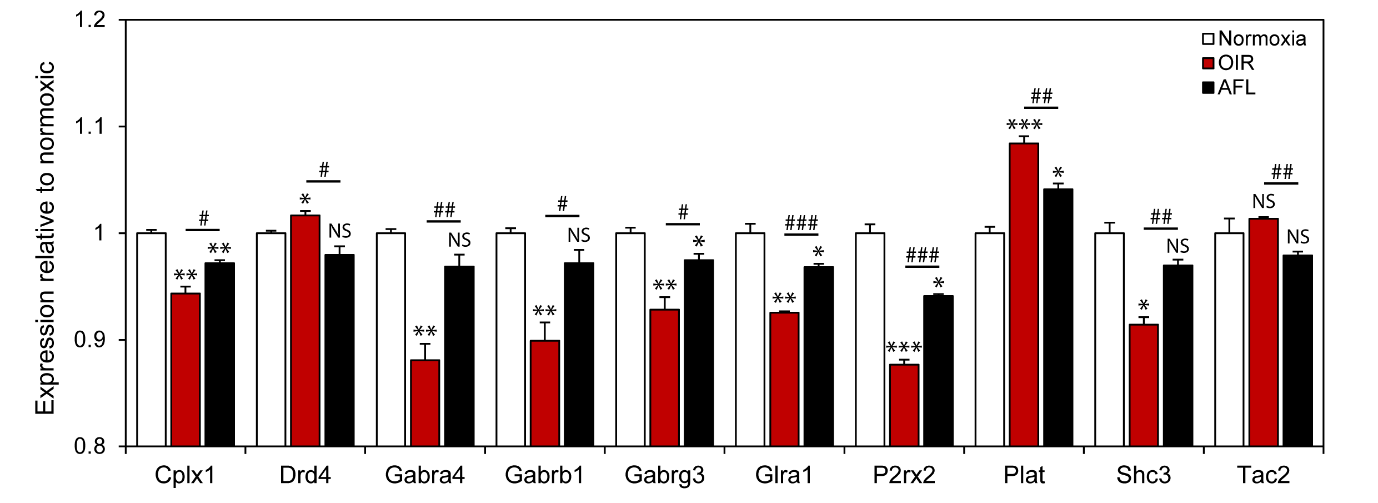


**Supplementary Figure 2.** Expression levels of genes contributing to the enrichment of the GO term “Chemical synaptic transmission” in the list of DRGs detected by comparing the transcriptional profile of P17 OIR+AFL and OIR retinas. Expression is shown normalized to the levels detected in normoxic controls. Statistical significance in comparisons relative to normoxia is denoted as follows: **P*<0.05; ***P*<0.01; ****P*<0.001. Additionally, statistical significance in comparisons between OIR and OIR+AFL samples is denoted as follows: ^#^*P*<0.05; ^##^*P*<0.01; ^###^*P*<0.001. NS, non-significant. Error bars depict +1 S.E.M.
